# Marine-Derived Polymers–Polysaccharides as Promising Natural Therapeutics for Atherosclerotic Cardiovascular Disease

**DOI:** 10.3390/md23080325

**Published:** 2025-08-12

**Authors:** Edmond Leonard Jim, Edwin Leopold Jim, Reggie Surya, Happy Kurnia Permatasari, Fahrul Nurkolis

**Affiliations:** 1 Department of Cardiology and Vascular Medicine, Faculty of Medicine, Sam Ratulangi University, Manado 95115, Indonesia; edmondleonardjim@unsrat.ac.id; 2 Department of Internal Medicine, Royal Taruma Hospital, Jakarta 11470, Indonesia; 3 Department of Food Technology, Faculty of Engineering, Bina Nusantara University, Jakarta 11480, Indonesia; 4 Department of Biochemistry and Biomolecular, Faculty of Medicine, Universitas Brawijaya, Malang 65145, Indonesia; 5 Master of Basic Medical Science, Faculty of Medicine, Universitas Airlangga, Surabaya 60131, Indonesia; 6 Institute for Research and Community Service, State Islamic University of Sunan Kalijaga (UIN Sunan Kalijaga), Yogyakarta 55281, Indonesia; 7 Medical Research Center of Indonesia, Surabaya 60286, Indonesia

**Keywords:** marine polysaccharides, atherosclerosis, cardiovascular disease, natural therapeutics, fucoidan, laminarin, drug delivery

## Abstract

Atherosclerotic cardiovascular disease (ASCVD) remains a leading cause of morbidity and mortality worldwide, driven by dyslipidemia, chronic inflammation, oxidative stress, and endothelial dysfunction. Despite widespread use of lipid-lowering and anti-inflammatory agents such as statins, residual cardiovascular risk and adverse effects underscore the need for novel, safe, and multi-targeted therapies. Marine-derived polysaccharides (MDPs)—including fucoidan, alginate, laminarin, carrageenan, and chitosan—exhibit a spectrum of bioactivities relevant to ASCVD pathogenesis, such as anti-inflammatory, antioxidant, lipid-modulatory, antithrombotic, and endothelial-protective effects. In this critical review, we synthesize preclinical and emerging clinical evidence on the pharmacokinetics, mechanisms of action, and therapeutic potential of these compounds. We highlight translational challenges, including structural variability, poor oral bioavailability, and limited human data, and propose strategies to overcome these barriers, such as molecular standardization, novel delivery systems, and well-designed clinical trials. MDPs represent promising natural therapeutics for ASCVD prevention and treatment, warranting further investigation in rigorous human studies.

## 1. Introduction

Atherosclerotic cardiovascular disease (ASCVD)—manifesting as coronary artery disease, cerebrovascular disease, and peripheral artery disease—is a principal contributor to global morbidity and mortality [1,2]. The pathogenesis of ASCVD is complex, involving lipid accumulation in the arterial intima, chronic vascular inflammation, oxidative stress, endothelial dysfunction, and thrombotic events [3]. Although statins and other pharmacological agents have transformed ASCVD management by lowering circulating low-density lipoprotein cholesterol (LDL-C) and attenuating inflammation, residual risk persists and may be compounded by adverse effects, interindividual variability, and poor long-term adherence [4]. Common side effects such as myopathy, liver enzyme abnormalities, and gastrointestinal discomfort can limit patient tolerance, while not all individuals respond equally to these therapies [5]. These limitations underscore the need for alternative or complementary treatments such as marine polysaccharides (MDPs), which offer promising anti-inflammatory and lipid-lowering properties with potentially fewer side effects in the management of atherosclerotic cardiovascular disease.

In light of these unmet needs, attention has turned to natural products as sources of novel, multi-targeted therapeutic agents [6,7]. The marine environment harbors an extraordinary diversity of polysaccharides—such as fucoidan from brown seaweeds, carrageenan from red algae, alginate and laminarin, ulvan from green seaweeds, as well as chitosan derived from crustacean shells—that display potent bioactivities relevant to ASCVD prevention [8,9]. These include suppression of pro-inflammatory cytokines, scavenging of reactive oxygen species, modulation of lipid metabolism, inhibition of platelet aggregation, and protection and repair of endothelial cells.

Despite promising preclinical findings, translation into clinical practice faces several challenges, such as polysaccharide structural heterogeneity, inconsistent extraction and purification methods, poor oral bioavailability, and scarce human data on safety and efficacy [10]. To unlock their therapeutic potential, rigorous structure–activity characterization, molecular standardization, advanced delivery platforms (e.g., nanoparticles), and well-powered, placebo-controlled clinical trials are required.

This review critically evaluates current knowledge on the bioactivities, pharmacokinetics, and mechanisms of action of MDPs in ASCVD models. We also examine translational barriers and propose future research directions to facilitate their development as safe, effective, and sustainable natural therapeutics for atherosclerotic cardiovascular disease.

## 2. Marine Polysaccharides: Natural Sugar-Based Bioactives

MDPs constitute a wide variety of naturally occurring sugar-based bioactives and have received attention for potential medicinal use in disease prevention and treatment [8,11]. The purpose of this section is to explore the sources, structural characterization, and biological activities of MDPs and how they are of importance for the prevention and treatment of cardiovascular diseases.

### 2.1. Definition and Classification of Marine-Derived Polysaccharides

**Figure 1:**
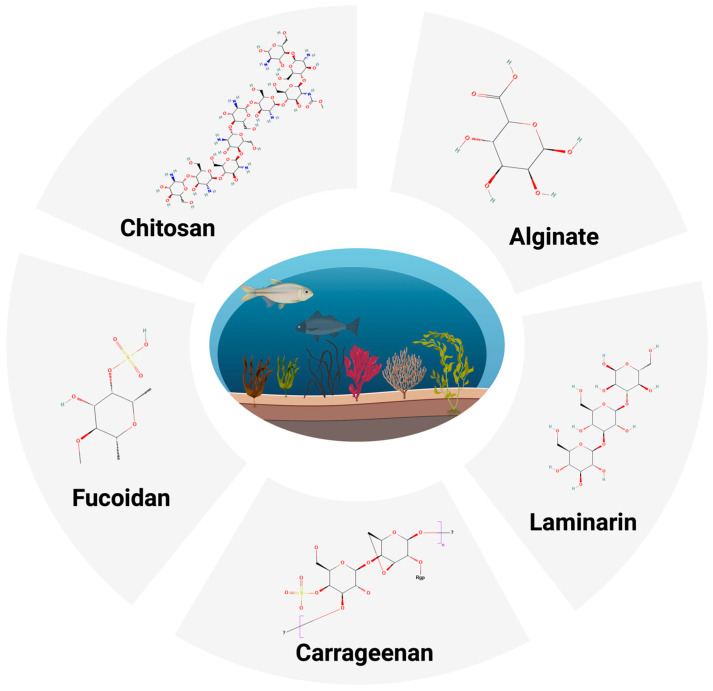
Structural diversity of marine-derived polysaccharides. Representative molecular structures of key marine polysaccharides, including fucoidan (highly sulfated), alginate (mannuronic and guluronic acids), laminarin (β-glucan), carrageenan (sulfated galactan), and chitosan (deacetylated chitin). Structural variations such as degree of sulfation and acetylation affect bioactivity.

MDPs are a chemically and structurally diverse group of natural polymers, encompassing fucoidan, alginate, laminarin, carrageenan, ulvan, and chitosan (Figure 1) [12]. These polysaccharides are distinguished by their monosaccharide composition, their glycosidic linkages, and sulfation or acetylation. Each of these chemical differences significantly affects their pharmacological activity. For instance, the highly sulfated structure of fucoidan enhances its immunomodulatory and anticoagulant effects, while the acetylated amino groups in chitosan are critical for its lipid-lowering and antimicrobial properties [13]. The high density and specific positions of sulfate groups in fucoidan lead to its strong negative charge, enabling effective interactions with positively charged components in the immune system and coagulation cascade, thereby enhancing its immunomodulatory and anticoagulant effects. Conversely, the presence of protonated acetylated amino groups in chitosan at physiological pH allows it to bind to negatively charged molecules like fatty acids and microbial cell membranes, facilitating its lipid-lowering and antimicrobial properties, respectively. This structural variance demands analytical methods to determine their exact structure and activity before they can be used in any clinical application [14].

The molecular architecture of MDPs profoundly influences their biological activities [15]. Variations in monosaccharide sequence, branching patterns, and levels of polymerization may significantly affect anti-inflammatory, antioxidant, and anticoagulant activities. For example, differences in branching patterns or the degree of sulfation in fucoidan greatly influence the differences in its bioactivity [14]. This underscores the complexity and promise of MDPs for biological applications [13,14]. Therefore, understanding the correlation of structure–activity is a critical aspect of harnessing their bioactivity.

MDPs are multifunctional in nature, with many exhibiting multiple pharmacological properties [16]. Fucoidan, for instance, has been demonstrated to have both anticoagulant and immune-modulating activities, while alginate is widely known for its utility in tissue engineering and drug delivery applications [17]. These multifunctional properties are critical in making these compounds suitable for multi-targeted therapies in complex conditions such as atherosclerosis. The differences in activity make it necessary to modify extraction and application conditions for specific applications.

Brown, red, and green seaweeds, crustaceans, and some marine microalgae are primary sources of MDPs [18]. Seaweed-derived fucoidan and laminarin are extracted from brown algae, whereas carrageenan is derived from red algae, and chitosan is commonly extracted from crustacean shells [19]. External factors play a pivotal role in determining their structure and bioactivity [20]. The marine environment, where these sources thrive, is a major factor that affects the structural features and cost-effectiveness of MDPs. Brown seaweeds are renewable sources that are widely available and economically suitable for fucoidan extraction, due to being globally abundant and extensively cultivated, particularly in Asia, thus increasing market demand for fucoidan’s diverse bioactivities. Also, utilizing crustacean shells, a seafood industry waste product, is cost-effective for chitosan extraction [13,19].

Due to the chemical variance of MDPs caused by intra- and interspecies variance in source and variability in extraction techniques, purity and consistency may affect their bioactivity, clinical applications, and other functionalities [17]. Genetic variability and varying extraction conditions can significantly affect their composition and biological activity [21]. For example, fucoidan from *Fucus vesiculosus* often has a simpler, more linear structure with distinct sulfation compared to the highly branched and varied structures found in *Sargassum* species, leading to differing anticoagulant or immunomodulatory potencies [22]. Meanwhile, extraction conditions like temperature, pH, solvent type, and duration can cause chemical alterations such as desulfation or depolymerization, directly impacting the final structural integrity and biological activity of fucoidan; for instance, high temperatures and acidic conditions during extraction can degrade fucoidan, reducing its molecular weight and thus its anticoagulant capacity [23]. This biological activity can, in turn, lead to issues with standardization and molecular impurity, and can thus hinder potential biomedical or therapeutic applications. Due to these variations, the molecular purity and consistency of MDPs, such as fucoidan, laminarin, alginate, carrageenan, and chitosan, may have significant differences [17]. To resolve this hurdle, molecular standardization techniques are crucial for preclinical and clinical assessment of MDPs, and could help promote consistency and efficacy.

The functional classification of MDPs underscores their therapeutic potential in various biological systems. Fucoidan and carrageenan are generally categorized as anticoagulant and immunomodulatory agents [24,25]. In contrast, alginate and chitosan, for example, are generally regarded to be effective for use in cellular scaffolding and gastrointestinal applications due to their excellent physicochemical properties [17]. Their various potential applications include wound healing, the prevention of cardiovascular diseases, drug delivery, etc. They may also be applied in the form of nanoparticles to enhance the delivery, stability, and activity of MDPs. These polysaccharides are also multifunctional, meaning that they could be useful in multiple applications. Some of the polysaccharides may function as drug delivery carriers and excipients in the drug development phase. Thus, MDPs have combined bioactivity and biological function [17]. Identifying appropriate MDPs to be applied for each clinical application is very crucial for optimizing drug delivery pipelines [19].

For clinical-scale development, one challenge is the standardization of MDPs. Slight differences in the molecular structure such as branching or sulfation degree significantly influence therapeutic activity. Different bioactivities can occur within small variances in structure, emphasizing the need for stringent and robust structure–function analysis for clinical efficacy [14]. However, these differences and variability allow for the discovery of structurally diverse scaffolds of bioactivity with the potential for use as the next generation of therapeutics [13,14]. This means that even minor variations in the position of a sulfate group on a fucose backbone can alter how strongly a fucoidan interacts with specific immune receptors, changing its anti-inflammatory potency.

Another barrier to the clinical assessment of MDPs lies in quality control. Due to the variations in the MDP-producing source, their crude preparations commonly yield inconsistent molecular masses. This has serious implications, as different production and extraction methods can alter the composition, bioactivity, and purity of the compounds that influence their in vivo responses. Consequently, clinical trials require robust quality control and molecular analysis as different batches might have major disparities in terms of activity and safety. Even trace amounts of impurities can elicit significant alterations in the bioactivity of MDPs [13]. Additionally, MDP variability affects the reproducibility and comparison of research findings. The lack of standardization and molecular classification of novel MDPs is one barrier to their scaled-up production and clinical usage for novel polysaccharides with clinical importance [19].

Thus, to fully optimize MDPs for therapeutics in diseases like atherosclerosis, more sophisticated and standardized clinical trials are required, along with better-controlled preclinical studies. To address challenges in development, a collaborative research methodology combined with technologies such as high-throughput screening, bioinformatic data analysis, and gene editing will assist marine scientists in improving the identification and optimization of MDPs to promote their application in complex diseases [17]. In particular, the careful assessment of the relationship between structural variations and bioactivity should be prioritized to facilitate the successful development of MDP-based interventions [14]. MDPs can be made with structural diversity, making them very valuable for therapeutic interventions [13,14,17,19].

### 2.2. Sources of Marine Polysaccharides

MDPs originate from multiple species, implying their ecological diversity. They can be extracted from brown, red, and green seaweeds, microalgae, and crustaceans [13]. Brown algae are one of the most abundant sources of fucoidan and laminarin from *F. vesiculosus* and *L. japonica* [13]. They are highly sulfated polysaccharides known for their anti-inflammatory and antioxidant activity. The most abundant polysaccharides found in red algae are carrageenans, which have outstanding gelling and immunomodulatory characteristics. They are extracted from species such as *Chondrus* and *Eucheuma* [26]. Chitosan comes from chitin deacetylation, which can be extracted from crustacean shells such as shrimp and crab [26]. The effect of lowering lipids, as well as its biocompatibility, contributes to chitosan’s importance for biomedical applications. Ulvan, a heteropolysaccharide, is notably derived from green seaweeds, such as *Ulva pertusa*, which is consumed as a marine vegetable in Asia [27]. Therefore, selecting the proper source of MDPs is essential for determining parameters such as sulfation patterns, molecular weight, and side chains, which ultimately have implications for the biological activities of atherosclerosis [17]. For example, the specific type of carrageenan (kappa, iota, or lambda) extracted from red algae determines its gelling strength and whether it activates or inhibits specific immune pathways [28].

The physiological effects of the ocean’s physical components such as temperature, ocean salinity, sunlight, and the availability of nutrient sources can have an impact on the composition and structure of the extracted MDPs. These parameters will significantly affect biological activity and lead to variable polysaccharide efficacy. Therefore, individuals belonging to the same marine species can possess differences in their biochemical compositions [17]. For example, fucoidan extracted from *Laminaria* harvested in cold waters contains a higher amount of sulfation, which leads to higher immunomodulation and anticoagulant effects compared to fucoidan extracted from *Laminaria* harvested in tropical regions [13]. Hence, the effects of geographical regions and climates can influence the bioactivities of MDPs and cause issues in standardized use.

Microalgae serve as an untapped resource for obtaining polysaccharides. *C. mexicana* and other species allocate up to 25% of their total organic production toward polysaccharides. These levels surpass all known macroalgae and crustacean resources [26]. Since biological activity varies drastically among different species, it is also critical to establish the exact source of these polysaccharides [17]. Microalgae produce polysaccharides in controlled conditions using a variety of biochemical pathways, resulting in structurally unique biomolecules and biological activities that are potentially superior to those from macroalgae or crustaceans. Aside from having beneficial effects on functional food and pharmaceutical applications, the controlled cultivation of microalgae enables year-round production without the significant negative impacts of harvesting in the ocean, where the biochemical components differ across seasons, geographical locations, and environments. Nevertheless, microalgae-derived polysaccharides need further extensive investigations to assess their potential use for medical purposes and to elucidate the structure–activity relationship [26].

MDPs exhibit a large range of biological activities, potentially due to differences in sulfation or in structural differences in terms of monosaccharide composition, molecular mass, or side chains. Therefore, each polysaccharide can possess distinct physiological activity and specificity with regards to atherosclerosis. Differences in their molecular structure can contribute to the specificity in certain targets, such as endothelial cells or certain inflammatory pathways through their capacity to modify levels of pro-inflammatory cytokines [13,17]. Hence, this variation demands not only the verification of the source but also their specific molecular profiling to ensure consistency of the pharmacological activities and bioactivities of the extracted polysaccharides. Different patterns of sulfation on fucoidan can lead to selective binding to specific chemokines, thereby influencing the recruitment of immune cells involved in atherosclerotic plaque formation [29]. Therefore, it is also important to identify and determine which specific features among these complex polysaccharides are responsible for certain therapeutic effects; this has not yet been extensively investigated in atherosclerosis [13].

The extraction and purification steps are critical since they have an impact on pharmacological effectiveness. One example is the use of alkaline or enzymatic hydrolysis to extract chitosan from crustaceans [26]. Although the composition is very similar to chitin, chitosan exhibits distinct biological activities because it lacks the presence of N-acetyl side chains [26]. In the case of seaweed-derived polysaccharides, extraction is commonly achieved through aqueous extraction and separation via fractionation [26]. Therefore, one cannot ensure consistency in the efficacy of these extracted molecules since these methods can alter parameters such as molecular weight, molecular shape, and branching. Consequently, establishing efficient and optimized procedures is essential for successful extraction of active ingredients while maintaining the integrity of the extracted materials and keeping their therapeutic effect. Also, purification protocols need to be in place to avoid contamination along with the presence of non-saccharide components and immunogenic impurities.

## 3. Anti-Atherosclerotic Mechanisms of Action

**Figure 2:**
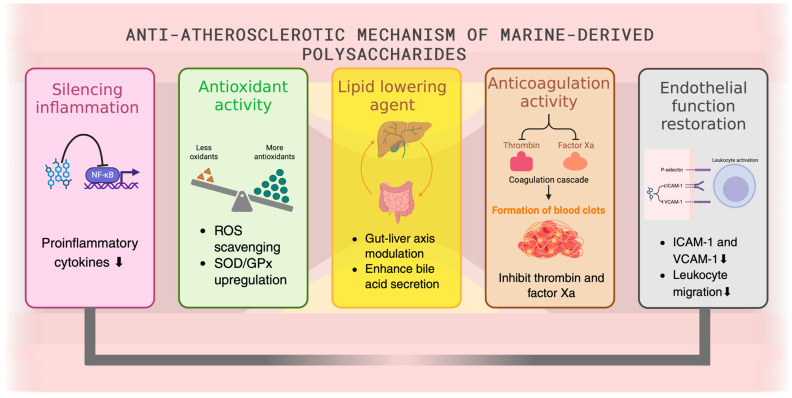
Mechanistic overview of anti-atherosclerotic activities. Schematic diagram illustrating the multi-targeted effects of marine-derived polysaccharides on key pathophysiological mechanisms of atherosclerosis: (1) inhibition of inflammation via NF-κB suppression, (2) antioxidant activity through ROS scavenging and upregulation of SOD/GPx, (3) lipid-lowering via modulation of gut–liver axis and bile acid excretion, (4) anticoagulation via inhibition of thrombin and Factor Xa, and (5) endothelial function restoration through reduced ICAM-1/VCAM-1 expression.

MDPs display several protective effects for cardiovascular health through various mechanisms affecting key pathological processes in atherosclerosis, including inflammation, oxidative stress, lipid levels, thrombosis, and endothelial dysfunction (Figure 2) [30]. Elucidating these mechanisms is key to developing effective treatments for cardiovascular disease; the following text describes these mechanisms in greater detail.

### 3.1. Anti-Inflammatory Effects

**Figure 3:**
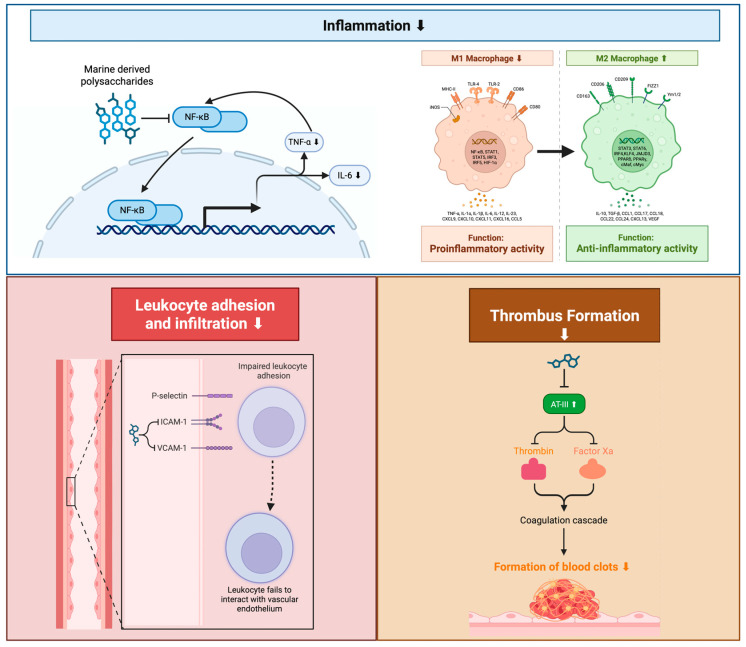
Effects of fucoidan on key inflammatory and coagulation pathways. Fucoidan inhibits IL-6 and TNF-α production, suppresses macrophage infiltration and adhesion molecules (VCAM-1, ICAM-1), and blocks thrombin-induced coagulation by enhancing antithrombin III activity. These actions contribute to the stabilization of atherosclerotic plaques and reduction in thrombotic risk.

MDPs display significant anti-inflammatory activity through the inhibition of pro-inflammatory cytokines and macrophages; these are factors that contribute to atherosclerosis development [31,32]. Fucoidan and laminarin downregulate key pro-inflammatory cytokines (i.e., IL-6, TNF-α) and macrophage infiltration in atherosclerotic plaques [33], thus suggesting that MDPs interrupt chronic inflammatory cycles that are crucial for atherosclerotic plaque development (Figure 3) [34]. By targeting cytokines that play a key role in both local and systemic inflammation, these natural products have the potential to disrupt the inflammatory processes of atherosclerotic plaque in a way that addresses multiple inflammatory mechanisms, unlike many anti-inflammatory therapies that target one specific inflammatory mediator or pathway.

Beyond cytokine modulation, fucoidan also actively reduces leukocyte adhesion and infiltration by downregulating adhesion molecules such as vascular cell adhesion molecule-1 (VCAM-1) and intercellular adhesion molecule-1 (ICAM-1) [35]. This action prevents leukocytes from interacting with the vascular endothelium and migrating into the arterial wall, which is a critical step in early atherogenesis and plaque progression (Figure 3). Furthermore, MDPs exhibit potent antithrombotic effects by inhibiting thrombus formation. Fucoidan, for example, enhances antithrombin III activity, which, in turn, blocks thrombin and factor Xa, both crucial components of the coagulation cascade [36]. These combined actions contribute to the stabilization of atherosclerotic plaques and significantly reduce the risk of thrombotic events.

By inhibiting IL-6 and TNF-α release in an experimental setting, MDPs decreased the activation of vascular and immune cells involved in atherogenesis [34]. Although this capability has been shown extensively in a wide range of studies, the ability of MDPs to inhibit IL-6 and TNF-α production in human models and/or in vivo clinical trials is unknown, along with whether or not such inhibition will lead to meaningful health outcomes in people at risk of cardiovascular diseases.

Ulvan, a sulfated polysaccharide extracted from green seaweeds, also exhibits significant anti-inflammatory effects by inhibiting the production of pro-inflammatory cytokines such as TNF-α, IL-6, and IL-1β [37]. It also suppresses the activation of the NF-κB and MAPK signaling pathways [38], thereby reducing inflammatory gene expression and mitigating immune cell recruitment to sites of vascular injury.

Several studies in animal models have shown that MDPs decrease macrophage infiltration into the arterial intima, a pivotal step in the generation of foam cells and the early stages of atherosclerosis [34,39]. By decreasing macrophage infiltration and its related inflammatory signals in the arterial wall, MDPs inhibit initial cellular events responsible for disease progression and reduce oxidative stress and lipid accumulation in plaque [34]. The mechanisms that govern the inhibitory action on macrophage infiltration are largely unexplored, particularly in human immunity and disease contexts. Future research is required to elucidate the mechanism through which select structural motifs of MDPs exert direct action on macrophage pro-inflammatory signaling to curb their inflammatory behavior and infiltration in human atherosclerotic plaques.

Additionally, MDPs regulate the phenotypic switch of macrophages, reducing M1 macrophages and promoting the differentiation and activation of M2 macrophages in the experimental atherosclerotic plaques [34]. As such, these natural products reduce inflammation and induce stability in atherosclerotic plaques, ultimately diminishing plaque development. MDPs target diverse molecular contributors of inflammation compared to several synthetic drugs that address the individual steps of these inflammatory responses [40,41]. This shift is crucial because M1 macrophages are pro-inflammatory and contribute to plaque growth, while M2 macrophages are involved in resolving inflammation and promoting tissue repair, thus stabilizing the plaque.

The anti-inflammatory potential of MDPs includes the inhibition of nuclear factor-kappa B (NF-κB) signaling, a main regulator of inflammation in atherosclerosis [42]. Activation of the transcription factor NF-κB enhances the expression of pro-inflammatory adhesion molecules, chemokines, and other signaling factors involved in the recruitment, adhesion, and transmigration of monocytes across endothelial and subendothelial matrix barriers to generate foam cells [43]. In one study, MDPs diminished the expression of VCAM-1 and ICAM-1 and decreased monocyte chemoattractant protein-1 (MCP-1) in experimental models of atherosclerosis [42]. All three factors are pivotal components of atherogenesis. Therefore, the ability of MDPs to interfere with the activation of these factors makes the use of natural marine products a promising anti-inflammatory therapeutic strategy for early-stage atherosclerosis, but requires further study into the long-term outcomes as well as off-target effects of NF-κB inhibition. By inhibiting NF-κB, MDPs directly reduce the production of these critical inflammatory mediators, thereby disrupting the cascade of events that lead to leukocyte adherence and infiltration into the arterial wall.

These MDP-dependent inhibitory events of NF-κB-mediated gene expression lead to a decrease in the expression of NF-κB transcription factor components as well as the downstream activation and expression of inflammatory factors in experimental models [42]. Specifically, MDPs suppress the transcription of key pro-inflammatory cytokines such as TNF-α, IL-1β, and IL-6, which are major mediators in the progression of atherosclerotic inflammation. Additionally, they reduce the expression of adhesion molecules such as VCAM-1 and ICAM-1, which facilitate leukocyte adhesion and transmigration into the vascular intima. Furthermore, inhibition of NF-κB reduces iNOS and COX-2, both of which contribute to oxidative stress and endothelial dysfunction. This finding is particularly significant due to the importance of NF-κB activation in controlling inflammation, which is an important mechanism in promoting the growth and pathogenesis of atherosclerotic plaques, as well as the development of vascular dysfunction [42]. Targeting NF-κB with MDPs may have added therapeutic benefits, particularly in clinical interventions where vascular inflammation is not adequately addressed using conventional pharmaceuticals. This activity requires further investigation in humanized experimental atherosclerosis models.

Structurally, MDPs possess a broad array of molecular configurations, which contribute to the varying anti-inflammatory activity of these naturally sourced substances [44]. For example, the degree of sulfation in MDPs is inversely correlated with their inhibitory potency against pro-inflammatory mediators [40,45]. This highlights the importance of molecular characterization for the design of promising treatments. As indicated above, highly sulfated polysaccharides are known to inhibit specific inflammatory mediators. Comparative assessments have revealed that modest differences in molecular conformation, which depend on extraction and purification methods, affect the functional ability of these compounds [46,47]. More comprehensive and uniform characterization of the structures of MDPs is therefore warranted to assure reproducibility and reliability for medicinal applications.

Structural characteristics such as molecular mass, monosaccharide composition, and structural branching contribute to their immune-modulatory profiles [40,45]. Despite the growing amount of evidence supporting the beneficial anti-inflammatory activity of MDPs in various experimental models, current data gaps limit the translation of these findings to human disease. Most investigations on this topic are predominantly limited to rodent models or in vitro research that do not capture the complex immune and vascular microenvironment of human atherogenesis [34]. Current information does not encompass human disease progression, and less information is known regarding the impact on other subsets of immune cells (i.e., dendritic cells and T-lymphocytes) involved in atherosclerosis. Additional studies on specific marine polysaccharide structures on these cell types and their roles in the complex immunoregulatory network governing atherosclerotic plaques in humans are needed [42].

There is a serious need for studies that investigate humanized experimental designs of disease, including clinical trials, to provide insights on the applicability of the anti-inflammatory activity of MDPs to individuals at risk of or afflicted with cardiovascular diseases. It is essential to determine how specific doses of structurally characterized MDPs affect humans and how specific biomarkers can be used to measure the therapeutic effectiveness of these natural marine products. Additional clinical trials will allow for more rigorous evaluations of efficacy, drug target pathways, and side effects. These translational efforts are imperative for utilizing the anti-inflammatory activities of MDPs in medical treatments. This involves identifying specific biomarkers, such as changes in circulating pro-inflammatory cytokines like IL-6 or TNF-α, that accurately reflect the anti-inflammatory effects of MDPs in human patients with atherosclerosis.

### 3.2. Antioxidant Properties

Several preclinical studies demonstrated the antioxidant properties of fucoidan, chitosan, and ulvan., Their antioxidant activities may scavenge free radicals, inhibit lipid peroxidation, and enhance the activity of endogenous antioxidant enzymes such as superoxide dismutase (SOD) and glutathione peroxidase [48,49,50,51]. Additionally, the biochemical role of MDPs and their oxidative status has been observed in vivo. Supplementation with MDPs significantly reduced oxidative status. Fucoidan, for example, reduced oxidative damage, evidenced by reduced lipid peroxidation and elevated antioxidant enzyme activities in animal models. These beneficial biochemical effects provide a basis for the hypothesis that MDPs, acting as antioxidants, prevent oxidative-stress-induced vascular damage to delay the occurrence of early-stage atherosclerosis [52,53]. However, translating these positive findings into a clinical context requires further investigation.

Interestingly, experimental results show that MDPs improved myocardial and vascular functions, protecting against oxidative damage. Fucoidan supplementation, for example, reduces lipid peroxidation activity, which leads to enhanced antioxidant profiles for myocardial and vascular injury [53]. However, since animal models of myocardial infarction and vascular injury do not totally mimic the pathological events occurring in humans, the ability of MDPs to ameliorate these diseases in humans remains to be examined in future studies.

Moreover, the antioxidant mechanisms exerted by MDPs also protect against the oxidative modification of biomolecules and the development and progression of atherosclerotic plaques. They scavenge ROS directly, and their influence on antioxidant enzyme expression improves the endogenous antioxidative defense system, resulting in diminished oxidative modification of lipids and other biomolecules. This characteristic presents a superior advantage over the effectiveness of a wide array of commercial antioxidants in rat models, since MDPs can work in a more multifaceted way [34,54]. This multifaceted action allows MDPs to both directly neutralize free radicals and bolster the body’s own antioxidant defenses, offering comprehensive protection against oxidative stress. However, the molecular basis for the antioxidant action of MDPs must be further clarified.

Sulfation in MDPs is a crucial factor that impacts its antioxidant properties. Numerous studies suggest that both the sulfate group and the monosaccharide residues have a crucial impact on the antioxidant properties of polysaccharides as [34,55,56]. For example, a higher degree of sulfation has been reported to enhance the antioxidant properties of fucoidan compared to the less-sulfated samples. This suggests the need for detailed molecular characterization in the extraction and preparation of MDPs used for antioxidant activities. The branching patterns and ratio of uronic acids also influence the antioxidant characteristics of MDPs. These structural characteristics suggest that polysaccharides can be selectively extracted from a particular organism and/or altered by chemical modifications in order to create selective antioxidant activity for functional or clinical applications [34]. Thus, the standardization of MDPs in clinical formulations is crucial, requiring effective analytical approaches. The increased negative charge introduced by a higher degree of sulfation can enhance the ability of fucoidan to interact with and quench positively charged free radicals, boosting its antioxidant capacity.

Polysaccharides isolated from the abalone viscera and fish oil decreased the malondialdehyde (MDA) content (a key marker of lipid peroxidation), but increased SOD activity in rodents, suggesting that polysaccharides help prevent lipid peroxidation and enhance antioxidant enzymes to promote vascular health [52]. Therefore, MDPs might be a good candidate to incorporate into functional food or nutraceutical-based products to prevent lipid peroxidation and elevate antioxidant enzyme activity [57]. Lipid peroxidation of low-density lipoprotein (LDL) contributes to foam cell formation, plaque instability, and atherothrombosis. It is a key event that initiates and promotes atherosclerosis. It has been suggested that MDPs may prevent LDL oxidation; therefore, they can be used in the prevention and treatment of atherosclerosis [53,54]. However, the inhibitory mechanism of MDPs in the oxidation of LDL still needs further elucidation.

Preclinical investigations have shown that the beneficial antioxidant properties of MDPs can protect endothelial cells. Oral intake of these polysaccharides can boost the expression of endothelial antioxidant enzymes and suppress endothelial cell apoptosis in cases of vascular injury [58,59]. Therefore, MDPs might have clinical benefit as comprehensive vascular protectants, preventing or ameliorating vascular events such as atherosclerosis, a disease which leads to endothelial dysfunction. This protective effect on endothelial cells is crucial because a healthy endothelial lining is the first line of defense against vascular injury and the initiation of atherosclerotic plaque formation.

To date, there is a lack of clinical investigations on the antioxidant activities of MDPs in enhancing vascular health. In addition, interpretation of the limited evidence available remains challenging due to various factors, including diversity in polysaccharide sources, extraction methods, dietary habits, and the limited number of human studies. More high-quality and adequately powered human clinical trials are required to elucidate and confirm the clinical antioxidant activities and effectiveness of MDPs in cardiovascular disease prevention (CVD).

### 3.3. Lipid-Lowering Potential

The lipid-lowering potential of MDPs has been assessed in preclinical studies through changes in plasma lipid profiles. For example, chitosan-oligosaccharides were shown to effectively lower TC and enhance HDL-C levels in hypercholesterolemic animal models, indicating their potential in treating dyslipidemia as a leading risk for atherosclerosis [52]. However, there is still a need to further investigate the molecular mechanisms of the lipid-lowering effect of MDPs, particularly in comparison with the mechanism of statins.

Furthermore, the lipid-modulatory effects of chitosan-oligosaccharides appear to be mediated through the regulation of the gut microbiota by increasing the number of Bacteroidetes [60,61]. The impact of gut–liver interaction on the metabolism of lipids by MDPs in vivo, however, has not been clearly explained. Future studies will need to explore whether MDPs can improve the ability of the gut microbiota to regulate lipid metabolism in humans, as well as whether MDPs can be combined with dietary fibers or probiotics to synergistically enhance the regulation of the gut–liver lipid metabolism. This increase in Bacteroidetes could contribute to lipid-lowering potential by influencing the production of short-chain fatty acids, which, in turn, can impact hepatic cholesterol synthesis and fatty acid oxidation.

Polysaccharides from seaweeds, such as those obtained from abalone visceral extract, brown algae, and green algae, have shown to lower serum TC, TG, LDL-C, and increase HDL-C levels, suggesting their potential to alleviate hyperlipidemia in hypercholesterolemic animal models [9,52]. In addition, polysaccharides from abalone visceral extract were found to decrease MDA content and increase SOD (superoxide dismutase) activity. The antioxidant activity of abalone visceral polysaccharide may provide more health benefits in reducing the risk of atherosclerosis because its ability to lower cholesterol levels is combined with antioxidative stress effects [62,63]. These polysaccharides must be investigated further in humans to ascertain if they are effective and whether they have any side effects.

Another potential mechanism of the lipid-lowering effect of MDPs may be by increasing cholesterol catabolism. In one in vivo study, ulvan was shown to improve cholesterol catabolism by increasing bile acid excretion, indicating that this polysaccharide is involved in the metabolism of cholesterol [53]. MDPs act in a different manner from the common cholesterol-lowering drug statin that inhibits cholesterol absorption. Ulvan may suppress cholesterol by promoting bile acid synthesis and excretion, resulting in reduced plasma cholesterol and hepatic cholesterol pools, and thus alleviating the atherogenic dyslipidemia associated with hypercholesterolemia [64,65]. While such findings are encouraging, future studies need to explore the effect of ulvan on cholesterol metabolism in clinical trials.

Chitosan and other polysaccharides from seaweed are known to lower plasma LDL-C and to increase HDL-C levels in both normocholesterolemic and hypercholesterolemic animals [53]; sometimes, they are even more effective than other lipid-lowering medicines, such as statins and probucol, in the prevention of atherosclerotic cardiovascular disease [34]. However, clinical trials must be completed to ascertain whether MDPs are effective in humans for regulating lipid metabolism, and if they have any side effects. The supplementation of fish-oil-derived or chitosan-oligosaccharides in rats led to downregulation of hepatic genes of the fatty acid synthesis pathway, providing evidence of a mechanism that can be used to lower serum lipid profiles [52]. Modulation of the gut microbiota by polysaccharides also seems to reduce hyperlipidemia in animal models, indicating the potential importance of polysaccharides on lipid metabolism regulation. The downregulation of hepatic genes involved in fatty acid synthesis suggests that these MDPs directly interfere with the liver’s ability to produce lipids, contributing to their serum-lowering effect.

Due to MDPs being regarded as safe in experimental investigations, as well as the long history of utilizing seaweed as a major food in traditional diets, MDPs show potential as functional foods [34,66]. Different MDPs have shown different anti-atherosclerotic effects in both cell and animal experiments. This difference may result from the purity of polysaccharide preparations as well as their structural differences. Therefore, it is vital to purify and optimize the specific extraction method of different MDPs. Also, the structures of the purified polysaccharides should be further elucidated to establish the relationship between the structure and biological activities, thereby enabling the discovery and development of marine polysaccharide-based drugs to treat hyperlipidemia with more reproducible efficacy.

Overall, the potential application of MDPs as lipid-lowering agents is very promising. In spite of the many efforts that have been made so far, there is an urgent need to further investigate more MDPs and clarify the mechanisms of their lipid-lowering effects in order to facilitate the future development of marine polysaccharide-based drugs for CVD management.

### 3.4. Antithrombotic and Anticoagulant Activity

Fucoidan and carrageenan extracted from seaweed exhibit promising antithrombotic properties in vitro by inhibiting platelet aggregation and reducing fibrin clot formation, thus reducing the risk of thrombotic events which can trigger an acute phase of atherosclerotic cardiovascular disease [67]. Ulvan extracted from *Ulva rigida* has also shown anticoagulant activity stronger than Lovenox^®^ and can be an alternative to heparins [68]. Fucoidan and sulfated polysaccharides block platelet activation by blocking calcium release mobilization within the platelet and inhibiting binding to von Willebrand factor and adhesion molecules [69,70]. These pathways provide antithrombotic potential. Fucoidan and sulfated polysaccharides have also been shown to block thrombin-induced clot formation [71,72]. In atherosclerosis, in which acute thrombosis contributes to vessel occlusion after plaque rupture, the use of such anticoagulants would minimize this outcome. Platelet activation can be inhibited through decreased intracellular calcium mobilization or by blocking ADP-induced platelet aggregation [73,74].

Fucoidan and sulfated polysaccharides also affect platelet adhesion. Platelet activation and adhesion in an atherosclerosis-mediated plaque rupture are important contributors of the acute thrombotic outcome. These properties would further diminish the atherosclerotic progression from plaque rupture to thrombotic events. In vitro fucoidan was also found to exhibit anticoagulant effects that are equal to, or surpass, those of heparin. These activities act by enhancing the function of antithrombin III. Fucoidan and sulfated polysaccharides inhibit factor Xa and thrombin and inhibit blood coagulation [42]. The degree of sulfation and the molecular weight of polysaccharides regulate their anticoagulant activity. Highly sulfated polysaccharides tend to have greater activity to inhibit coagulation, enhancing the antithrombin III-mediated inhibition of thrombin and factor Xa [9]. This mechanism of enhancing antithrombin III activity means that fucoidan essentially amplifies the body’s natural anticoagulant system, leading to a more potent inhibition of key clotting factors.

Fucoidan has also been observed to inhibit the activity of intrinsic factors involved in the initiation of blood coagulation [75]. One isolated fucoidan fraction exhibited higher activity for the inhibition of blood coagulation than low-molecular-weight heparin. The heterogeneity in anticoagulant activity among various fucoidans arises due to variations in molecular size or in extraction procedures [42]. The high sulfation of polysaccharides derived from brown algae results in the enhanced inhibition of platelet aggregation compared to lower-sulfated polysaccharides and results in higher anticoagulant activity. These observations clearly demonstrate the effect of molecular characteristics on platelet aggregation [9].

Not all preparations of polysaccharides show equivalent antithrombotic effects. For example, highly sulfated fucoidan inhibited platelet aggregation to a much greater extent than a fucoidan preparation with a significantly lower sulfation level [9]. Polysaccharides may contain batch-to-batch differences within and among polysaccharide preparations that alter their antithrombotic efficacy, limiting their practical application. Current approaches to overcome this problem include a process called “fractionation” along with characterization of the various structures of the extracted polysaccharides. Fractionation isolates and preserves the polysaccharide fraction that has a highly potent and repeatable anticoagulation effect [9]. This fractionation process is critical because it isolates the specific range of molecular weights and sulfation patterns within the crude extract that are most effective at inhibiting thrombosis, ensuring consistent therapeutic outcomes.

In vitro experimental data show that fucoidan and carrageenan are both antithrombotic because of their ability to reduce platelet activation and adhesion, resulting in inhibition of clot formation in the vasculature [9,67]. Platelet aggregation is a primary driver of clot formation in this scenario. The activation of platelets at the site of injury initiates a cascade of events that facilitates clot and fibrin formation. Adhesion molecule receptors are highly important in initiating and maintaining platelet activity and aggregation in clot formation and stabilization in the case of atherosclerotic lesion rupture. Fucoidan has been shown to reduce P-selectin expression, thereby minimizing platelet adhesion at the endothelium [9]. These polysaccharides block both primary and secondary hemostatic processes within vascular injury, leading to diminished clot formation. In the case of plaque rupture, inhibition of the primary stages of platelet aggregation and platelet adhesion, in combination with fibrin stabilization of the thrombus, contribute directly to the prevention of a complete thrombotic event with arterial obstruction [76].

Polysaccharides are polyvalent substances, as opposed to single target substances, which may allow for a broader inhibition of the platelet activation and stabilization pathways. Their polyvalence may lead to improved outcomes in comparison to the current therapeutic armamentarium, which targets single points of entry to inhibit platelet function [67]. Both of these polysaccharides appear to be relatively safe to consume orally on a regular basis. Studies have not reported the incidence of bleeding to be significant [9]. This polyvalency means that a single MDP can interact with multiple receptors or enzymes involved in coagulation and platelet function, offering a more comprehensive anticoagulant effect than drugs targeting only one specific pathway.

Despite its benefits, several critical limitations hinder the commercial availability of marine-derived polysaccharide therapeutics. These factors include variability in bioactivity between polysaccharide preparations, bleeding risks, the lack of a standard extraction method, and the inconsistent nature of purification techniques [42]. Another critical impediment to clinical translation is its poor oral bioavailability and inconsistent pharmacokinetics. The oral intake of polysaccharides induces gut microbiota changes that may impact its bioavailability and pharmacokinetic pathways [42]. Differences between species (animals) for the distribution and metabolism of these polysaccharides remain critical barriers to predicting their function and efficacy in human treatments, since the extrapolation of results from animal models to human subjects may not be applicable.

This requires the development of novel oral delivery systems and further pharmacokinetic and toxicity studies in humans, which would enable them to reach the plasma in efficacious levels; this would improve treatment against atherosclerotic cardiovascular disease and potentially contribute to decreasing the mortality and morbidity associated with the disease. Regulatory approval will depend on addressing issues such as standardization, analytical methods, and assessing the risk–benefit profile of these polysaccharide molecules, since the lack of a standardized extraction and purification method for these compounds has proven to be a major hindrance to the validation of its efficacy and safety to date [9].

The most promising research directions to move polysaccharide therapeutic benefits closer to practical applicability will be the development of a functionalized polysaccharide delivery system that ensures proper pharmacokinetic and bioavailability features of polysaccharide drugs for the oral route of delivery, the design and development of clinical trials addressing crucial research gaps, such as determining the optimal drug concentration, the identification of other biological properties and/or mechanisms by which polysaccharides exert their efficacy against atherosclerosis and platelet activation and/or aggregation, as well as studies addressing long-term use, safety, and efficacy in atherosclerosis management [42]. One such functionalized delivery system could involve encapsulating fucoidan within pH-sensitive nanoparticles, allowing its release only in specific parts of the intestine for improved absorption and targeted action [77].

### 3.5. Endothelial Function Restoration

**Figure 4:**
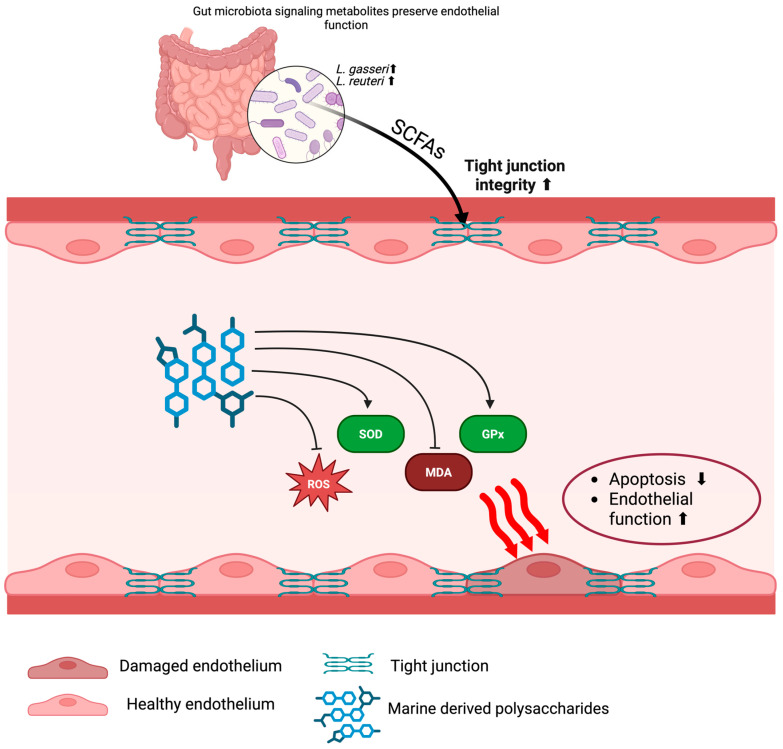
Marine polysaccharide interactions in endothelial repair and antioxidant defense. Marine-derived polysaccharides reduce oxidative stress markers (e.g., MDA) and enhance antioxidant enzymes (e.g., SOD, GPx), leading to preserved endothelial cell integrity, reduced apoptosis, and improved vascular reactivity. Gut microbiota modulation also contributes to endothelial homeostasis.

Fucoidan and ulvan have been demonstrated to improve endothelial function and delay atherosclerosis progression (Figure 4) [59,78,79]. Experimental evidence has suggested that the potency of fucoidan in improving endothelial cell function and repair may depend on molecular weight. For instance, lower-molecular-weight fucoidan (3.2 and 7.4 kDa) demonstrated more potent activity in improving endothelial cell viability and repair postinjury [80]. This enhanced activity is likely due to better cellular uptake, improved bioavailability, and greater interaction with surface receptors involved in endothelial regeneration, such as vascular endothelial growth factor receptor 2 (VEGFR2) and fibroblast growth factor receptors (FGFRs). In contrast, medium– high-molecular-weight fucoidan (≥ 15 kDa) has shown weaker effects on endothelial repair, possibly due to steric hindrance, limited permeability, and reduced binding efficiency to endothelial cells [81]. However, these studies indicate that high-molecular-weight fucoidan may still contribute to endothelial protection by exerting antioxidant and anti-inflammatory effects through inhibition of leukocyte adhesion and suppression of oxidative stress markers. However, clinical trials are lacking, and it remains undetermined whether the molecule size matters in the human system with regard to oral bioavailability. MDPs also exert protection on endothelial function via antioxidant mechanisms by reducing oxidative stress, oxidative biomarkers, and enhancing SOD and glutathione peroxidase (GPx) activities in the vascular system [34]. The reduction in oxidative stress helps prevent endothelial cell apoptosis and thus preserves vascular reactivity and the progress of atherosclerosis. However, the effects of these antioxidant pathways need to be examined in clinical trials.

The chemical properties of MDPs such as the degree of sulfation, molecule conformation, and arrangement affect their activity on vascular tissue, which remains poorly elucidated. For example, higher-sulfated fucoidan enhanced endothelial repair and reduced oxidative damage [34,80]. In both basic science and clinical trials of MDPs, the chemical properties should be well characterized. This will help improve and ensure the consistent quality of the materials, and thus reduce variability in efficacy and outcome. MDPs were also shown to preserve and restore endothelial function through anti-inflammatory effects. They have been reported to downregulate the expression of adhesion molecules ICAM-1 and VCAM-1 and inflammatory cytokines IL-6 and TNF-α, thus diminishing monocyte adhesion to the endothelium [9]. In summary, by preventing or improving endothelial inflammation via the anti-inflammatory mechanisms mentioned above, MDPs could effectively ameliorate endothelial function to restore the endothelium and delay atherogenesis. However, since most studies are preclinical, more research is warranted to ensure that the above anti-inflammatory effects are effective in human systems.

MDPs may be able to improve endothelial function through the gut-vascular axis by modulating the gut microbiome. MDPs have been reported to alter the gut microbiome, increasing beneficial strains such as Lactobacillus gasseri and Lactobacillus reuteri, resulting in an increase in short-chain fatty acids (SCFAs) to improve endothelial cell signaling and tight junction integrity [80]. Therefore, MDPs have the potential to be used systemically to benefit cardiovascular health. However, this effect requires more in-depth research on how changes in the gut microbiome affect endothelial cell functions in vivo. The increased SCFAs produced by beneficial gut bacteria can act as signaling molecules that strengthen the endothelial barrier, reducing its permeability and thus the infiltration of inflammatory cells and lipids [82].

Though MDPs have demonstrated the ability to improve endothelial function through the mechanisms mentioned above, limitations still exist when translating into the clinical field, mainly due to the lack of data in the human system for bioavailability, dosing, and long-term safety [9,34,80]. Variation in polysaccharide sources and extractions also hinders their consistent potency and efficacy in the clinical setting. Well-designed clinical trials are therefore warranted to evaluate the benefits and mechanisms of polysaccharides at treating or preventing cardiovascular diseases. In addition, technological advances in nano delivery systems could improve the efficacy of MDPs.

In conclusion, MDPs have demonstrated effectiveness in improving endothelial function through different mechanisms. They may also work synergistically or sequentially to improve endothelial cell survival and function. Future research should be prioritized in elucidating the precise mechanisms by which the gut microbiome, antioxidant system, inflammation and endothelium work synergistically, how the chemical and physical properties of the molecules could be manipulated and modified, and how the MDPs improve endothelial function through various interactions.

### 3.6. Supporting Evidence from Preclinical Studies

MDPs exert their multifaceted anti-atherosclerotic effects not only through broad pharmacological activities but also via the precise regulation of specific proteins and genes crucial to disease progression. In terms of anti-inflammatory actions, these polysaccharides, such as fucoidan, have been shown to inhibit the nuclear factor-kappa B (NF-κB) signaling pathway [83], a pivotal regulator of inflammation that controls the expression of numerous pro-inflammatory adhesion molecules, chemokines, and other signaling factors. This inhibition leads to a decrease in the expression of NF-κB transcription factor components and downstream inflammatory factors. Regarding lipid metabolism, MDPs, particularly fucoidan, influence hepatic gene expression. They have been observed to upregulate cholesterol α-hydroxylase, an enzyme which is critical for bile acid synthesis and cholesterol catabolism. Conversely, they can downregulate sterol regulatory element-binding protein-1c/2 (SREBP-1c/2) [84], master regulators of fatty acid and cholesterol synthesis, respectively. These specific molecular interventions underscore the nuanced regulatory capabilities of MDPs, offering targeted therapeutic potential in managing atherosclerosis.

Preclinical studies show that fucoidan, laminarin, alginate, carrageenan, and chitosan have been investigated for their ability to decrease atherosclerotic risk factors. Several in vivo studies have shown that MDPs improve lipid profiles in hypercholesterolemic and high-fat-diet-induced animal models [9,34]. In vivo studies in hypercholesterolemic and high-fat-diet-induced animal models have demonstrated that MDPs significantly improve lipid profiles. Specifically, in LDL receptor mice on a high-cholesterol diet, intragastric gavage with 100 mg/kg fucoidan significantly reduced atherosclerotic plaques in the aortic arch, descending thoracic aorta, and abdominal aorta [9]. Similarly, spontaneously hyperlipidemic mice fed a high-fat diet supplemented with 1% and 5% fucoidan for 12 weeks showed a notable reduction in atherosclerotic lesion area in their aortas [85]. These findings were further supported by a study where intraperitoneal administration of 50 mg/kg fucoidan every third day for 16 weeks significantly reduced lesion formation in the aorta of hyperlipidemic mice [84]. Lipid improvement is the key, as dyslipidemia plays a critical role in the development and progression of atherosclerosis. While animal studies display promising results, the effects of MDPs in lipid improvement mechanisms, such as cholesterol metabolism, bile acid excretion, and lipid circulation in humans, are not yet understood.

Apart from the beneficial effects in lipid improvement, preclinical studies have displayed that MDPs reduce atherosclerotic plaque development in animal models as compared to controls [9,86]. Plaque reduction also aligns with the anti-inflammatory, antioxidant, and lipid-lowering effects displayed preclinically. These effects are mediated through multiple mechanisms, including the inhibition of pro-inflammatory cytokine production (such as TNF-α and IL-6), the suppression of ROS generation, and the modulation of lipid metabolism by altering cholesterol absorption and promoting reverse cholesterol transport. While MDPs have demonstrated a positive impact on atherosclerotic risk factors in animal studies, their efficacy and benefit in human atherosclerosis is not known. Human atherosclerosis is a more complex phenomenon that also includes systemic inflammation, metabolic abnormalities, and endothelial dysfunction in addition to inflammation within the vessel wall.

MDPs also exhibit some lipid-lowering effects that are similar in potency to some commercially available pharmacotherapies with less risk of adverse side effects [34]. These compounds also contribute to endothelial protection by enhancing nitric oxide production and reducing endothelial adhesion molecule expression, thereby improving vascular function and reducing monocyte recruitment to lesion sites. In some studies, not all MDPs have shown efficacy in lipid improvement. These observations suggest that the variances in the beneficial effects may be due to the variances in polysaccharide extraction and processing, and even the specific species.

The mechanisms of MDPs for improving lipids include cholesterol catabolism promotion, bile acid excretion improvement, and the improvement of hepatic lipid metabolism [53]. Fucoidan alters lipid uptake and metabolism, reducing serum total cholesterol, triglycerides, and LDL levels while increasing HDL in animal models. Hepatically, fucoidan upregulates PPARα for fatty acid β-oxidation and downregulates SREBP1, decreasing lipid synthesis. It also downregulates SREBP2, impacting cholesterol synthesis and uptake [87]. These actions suggest fucoidan’s role in altering atherosclerotic plaque development by modulating lipid metabolism. Additionally, ulvan promotes cholesterol breakdown into bile acids and increases their excretion, thereby lowering serum cholesterol [88]. Carrageenan also contributes by binding bile acids and cholesterol in the intestines, leading to increased cholesterol utilization for bile synthesis [89]. However, there have not been any studies investigating which of these pathways contribute to which types of lipids, such as cholesterol, triglycerides, and LDL-C. The overall mechanisms of marine-derived polysaccharide bioactivity in relation to the atherosclerotic plaque reduction are not yet known in humans.

Fucoidan and chitosan, two prominent MDPs, have displayed anti-inflammatory and antioxidant effects by suppressing pro-inflammatory cytokines, inhibiting macrophage infiltration, and improving antioxidant enzyme activities SOD in various in vivo models [34,90]. Anti-inflammatory and antioxidant effects counteract the key driving factors of atherosclerosis, vascular inflammation, and oxidative stress. Some of the mechanistic pathways these bioactive compounds act on include NF-κB signaling; however, it remains to be seen how their direct or indirect actions impact the atherosclerotic plaque and vessel remodeling clinically. In a rat model study, MDPs such as fucoidan reduced the production of inflammatory markers TNF-α and IL-6. These findings suggest that MDPs may be able to improve vascular inflammation by decreasing key signaling pathways such as NF-κB [42]. However, there is limited understanding of how these signal pathways translate and affect humans in the development of plaque in vascular walls, as inflammation can differ due to genetics, environment, diet, and other variables.

MDPs contain potent antioxidant properties and have been shown to scavenge free radicals by upregulating SOD, reducing MDA, and decreasing oxidative lipid modification [34]. By preventing the formation of foam cells and suppressing oxidative stress, MDPs were able to inhibit the plaque deposition in the artery. However, the long-term effect on how antioxidants lead to changes in atherosclerosis has not yet been studied in humans. Animal models have shown significant improvements in vascular disease severity, as MDPs have reduced the progression of vascular lesions, improved vascular reactivity, and induced positive vascular wall changes [90]. All of these factors can lead to the reduction in overall vascular damage; however, animal models often have differences in genetic background, gut flora, and diet. More preclinical studies need to be conducted in vitro and in vivo to evaluate if these beneficial effects are equally displayed across species.

Ulvan and chitosan-oligosaccharides have exhibited positive effects on enhancing bile acid excretion and altering gut microbiome, leading to increased absorption, lower circulating lipids, and lower rates of atherogenesis [9,90]. These effects occur through increased binding of bile acids in the intestine, promoting fecal excretion and stimulating hepatic conversion of cholesterol to bile acids, thereby reducing plasma cholesterol levels. These interesting results have not been investigated and applied to humans, and it is still unclear how these findings relate to each other in lipid modulation. MDPs impact lipid-related gene expression by upregulating cholesterol 7α-hydroxylase and downregulating sterol regulatory element-binding protein-1c/2 in the liver [52]. However, these effects have not been directly explored for their implications in plaque remodeling, which leads to changes in plaque regression and instability. Future research is needed to understand the full potential of these effects in regulating lipid metabolism. Several MDPs are prebiotics, promoting the growth of beneficial gut flora, which leads to reduced systemic inflammation and improved lipid metabolism [90]. Though there has been research indicating their ability to manipulate gut microbes, the full potential of how these specific types of microbes lead to benefits in cardiovascular health needs more investigation.

MDPs have showed promising antithrombotic activity by decreasing platelet aggregation and reducing fibrin clot formation in rodent models [9,42]. This antithrombotic effect is partly mediated through the inhibition of thromboxane A2 synthesis and the downregulation of glycoprotein IIb/IIIa receptors on platelet surfaces, which reduces platelet activation and aggregation. Given that arterial thrombosis plays a crucial role in human atherosclerosis, MDPs are able to counteract and alleviate these effects for dual benefits to improve atherosclerosis. However, it has also been shown that some MDPs have displayed high thrombotic activities in both in vivo and in vitro studies depending on the molecular weight and sulfation pattern of polysaccharide chains. Because of the differences in the results, advanced standardization of structural validation of MDPs is still required for further investigation for their antithrombotic effects.

Sulfated MDPs fucoidan enhances antithrombin III activity and inhibits activated factor Xa, thrombin, and other activated coagulation enzymes [42]. This occurs through the interaction of negatively charged sulfate groups on fucoidan with positively charged domains of coagulation enzymes, mimicking the mechanism of heparin and facilitating conformational changes that inhibit enzyme activity. Because it possesses antithrombotic activity, fucoidan can be used for the treatment or prevention of thrombosis. Yet, studies have not fully explored how fucoidan inhibits thrombin. Some animal studies have displayed adverse effects of increased bleeding risks with high consumption of fucoidan. Further investigation is needed to elucidate the mechanistic interaction between the coagulation pathway and these MDPs for clinical application.

MDPs exhibit antioxidant and endothelial-protective activities, both of which have direct effects on blood vessel cells and have indirect actions on intestinal microbes, leading to changes in circulating gut-derived metabolites [91,92]. Mechanistically, these compounds scavenge reactive oxygen species and upregulate endothelial nitric oxide synthase (eNOS), thereby enhancing nitric oxide bioavailability and improving vascular tone and function. Alterations in the microbiome, with changes in microbiome diversity, is known to lead to improvements in endothelial function [80]. Yet, it has not been revealed if these changes in the gut microbiome lead to improvements in atherosclerosis. It is not fully understood whether the alteration of the gut microbial composition can also contribute to the improvement in lipid metabolism, or which exact changes in the microbial population lead to the improvements in the cardiovascular system.

The mechanisms and properties discussed lead to several advantages in improving cardiovascular health with MDPs. However, their poor and variable bioavailability in absorption of intestines due to the differences in composition and source in species, complex structures, and large molecular size leads to inconsistency. The lack of standardization and lack of proper extraction, isolation, and purification, and a lack of human clinical studies contribute to the inconsistent effects displayed by these MDPs [34,42,90]. Future studies should focus on developing encapsulation strategies for MDPs to enhance their bioavailability.

In conclusion, despite these aforementioned issues, there is robust data that supports the bioactivity of MDPs and their potential for the prevention or improvement of atherosclerotic risk factors and atherosclerosis itself. With these promising preclinical findings, further clinical research should be conducted and expanded upon to determine the effect of marine-derived polysaccharide consumption by humans on the prevention or mitigation of the progression of atherosclerosis.

## 4. Translational Barriers and Scientific Challenges

**Figure 5:**
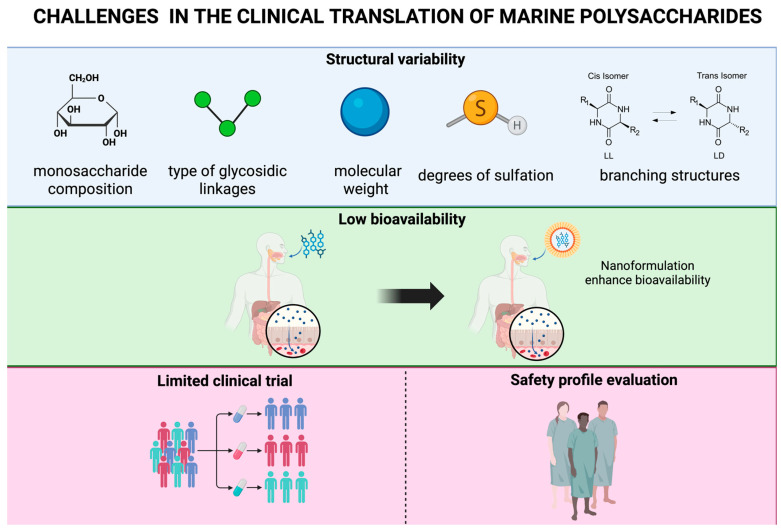
Translational roadmap for marine polysaccharides in ASCVD therapy. Challenges and strategies in the clinical translation of marine polysaccharides, including the following: (1) structural variability and need for standardization, (2) poor oral bioavailability and potential of nanoformulations, (3) limited human data requiring robust clinical trials, and (4) safety evaluation protocols for long-term use.

Bridging the gap between promising preclinical findings and effective clinical applications remains a major scientific and logistical challenge (Figure 5). The dearth of human data, bioavailability issues, structural variation, and safety concerns all demand more comprehensive investigations for MDPs to emerge as reliable therapies for atherosclerotic cardiovascular disease. Overcoming these barriers requires more elaborate and standardized investigations, novel technologies, and the willingness to further explore the therapeutic capabilities of these compounds.

### 4.1. Limited Clinical Data

The limited clinical data on the application of MDPs to atherosclerotic cardiovascular disease creates significant barriers to confidently moving these compounds into therapeutic applications. Of more than 100 preclinical studies examined, only 6 studies emerged which were related to ASCVD (Table 1). The majority of studies are in vitro or in animal models, making it difficult to extrapolate the efficacy and safety to human clinical populations [19]. There is an unmet need to conduct human trials to ensure the safety and efficacy of these compounds across the diversity and heterogeneity of cardiovascular patients around the world.

The provided clinical trials illustrate a varied but promising landscape for marine-derived polysaccharides in cardiometabolic health. Chitosan oligosaccharides and carrageenan show clear benefits in human lipid profiles, with carrageenan notably demonstrating significant reductions in total cholesterol and triglycerides, and an increase in HDL-C in human volunteers [93]. Chitosan oligosaccharides also exhibited broad improvements across lipid profiles, antioxidant capacity, and even left ventricular ejection fraction in CHD patients, alongside beneficial shifts in gut microbiota [94].

For fucoidan, while animal studies and some human trials suggest benefits, consistency can be an issue. A polyphenol-rich Fucus vesiculosus extract containing fucoidan is currently being investigated for its impact on LDL cholesterol and inflammatory markers in an ongoing study. Earlier trials with fucoidan have shown mixed results, with one study reporting no significant effect on insulin resistance or most cardiometabolic markers in obese, nondiabetic subjects, while another noted decreased diastolic BP and LDL cholesterol but increased insulin and worsened insulin resistance [95].

**Table 1:** Summary of clinical trials on marine-derived polymers and polysaccharides for atherosclerotic cardiovascular diseases.

Polysaccharide Type	Study	Design	Participants and Condition	Duration and Dose	Key Findings	Trial ID
Carrageenan (Red Algae)	[93]	Randomized Crossover Trial	20 healthy adults	8 weeks, ~40 g dietary fiber/day	↓ Total cholesterol (33%), ↓ triglycerides (32%), ↑ HDL (32%); no change in LDL	Not specified
Fucoidan (Brown Algae)	[95]	Double-blind RCT	72 obese, nondiabetic adults	90 days, 500 mg twice/day	↑ HDL (small, significant); no changes in HOMA or most markers; safe profile	ACTRN12614000495628
Polyphenol-rich Fucus vesiculosus Extract (Brown Algae, contains Fucoidan)	[66]	Double-blind RCT	58 overweight/obese with high LDL-C (>2.0 mmol/L)	12 weeks, 2000 mg/day (1200 mg fucoidan, 600 mg polyphenols)	Primary: LDL-C reduction (hypothesized); Secondary: lipids, glucose, insulin, inflammation, cognition	ACTRN12617001039370
SXRG84 (Ulvan) (Green Algae, *Ulva* sp.)	[96]	Double-blind RCT	64 overweight/obese adults (median BMI 29)	6 weeks, 2 g or 4 g/day	2 g: ↓ non-HDL (-10%), ↓ atherogenic index (-50%), trend in ↓ 2 h insulin; 4 g: ↓ CRP (-27%), ↑ beneficial gut flora	ACTRN12615001057572
SXRG84 (Ulvan) (Green Algae, *Ulva* sp.)	[96]	Double-blind Crossover RCT	64 overweight adults	6 weeks each (2 g/day SXRG84 and placebo)	No lipid differences; ↓ inflammatory cytokines: IFN-γ, IL-1β, TNF-α, IL-10; no gut flora shift	ACTRN12617001010381
Chitosan Oligosaccharides (Crustaceans)	[94]	Parallel-Group Clinical Study	120 CHD patients (60 per group)	6 months, 2 g/day	↑ LVEF, QOL; ↓ TG, TC, LDL-c; ↑ HDL-c; ↑ antioxidant markers (SOD, GSH); ↓ ALT, AST	Not specified
Palmaria palmata (Red Algae)	[97]	Double-blind Parallel RCT	104 Japanese with LDL-C ≥120 mg/dL	8 weeks, 2 g/day	No change in LDL-C, BMI, glucose; in women: ↓ TG and ↓ TG/HDL-C ratio (significant)	Not specified

↑ increase and ↓ decrease.

Ulvan from green seaweeds (SXRG84) has demonstrated significant anti-inflammatory effects by reducing CRP and other pro-inflammatory cytokines in overweight participants, even if its lipid-lowering effects were not consistently replicated across all studies [96]. The study also highlighted the prebiotic potential of SXRG84 through observable gut microbiota shifts. Finally, Palmaria palmata, a red algae, showed a significant reduction in serum triglycerides specifically in women [97], although it did not significantly affect LDL-C or glycemic control in the broader hypercholesterolemic cohort.

Overall, the evidence suggests that marine polysaccharides hold significant potential, but their efficacy can be influenced by the specific polysaccharide type, its molecular structure, the health status of the participants, and the study design. The varying outcomes underscore the need for more standardized and larger-scale human clinical trials to fully elucidate their therapeutic potential and establish clear guidelines for their use.

Currently, clinical data often suffers from low participant sample sizes, insufficient intervention durations, and inconsistent endpoint measures [19]. These factors can reduce statistical power and reproducibility in the study, making it difficult to validate the therapeutic effectiveness and safety of MDPs in humans. This may also hinder the ability to develop clinical consensus and guidance regarding the therapeutic use of these compounds in atherosclerotic cardiovascular disease. Larger clinical trials should be designed to address these shortcomings. Another concern regarding clinical trials is the varied control groups and clinical population included. This heterogeneity can reduce the ability to compare results across trials or perform meta-analysis [19]. Variability in participants can introduce concerns regarding the consistency in efficacy and safety between different clinical populations, particularly across populations that may have significant differences in genetics, diet, and exposure to environmental toxins. Therefore, a standardization of clinical trial design in terms of control group and participant population must be addressed. Additional large-scale, placebo-controlled, and multi-center trials need to be performed to validate the reported in vivo bioactivities (i.e., anti-inflammatory, antioxidant, lipid-modulatory, and endothelial-protective) of MDPs [9,34]. This would bring higher-quality data to be accepted into regulatory submission and support the regulatory approval and use of these MDPs in clinical therapy.

One challenge to clinical translation is difficulty tracking the pharmacokinetics, systemic absorption, and tissue bioavailability of MDPs, as existing detection methods lack the sensitivity to reliably and accurately determine systemic absorption efficiency or the resulting plasma levels for therapeutic benefit [98]. Therefore, it is difficult to measure the delivery, absorption, metabolism, and systemic clearance of these compounds within the human body. This would, in turn, make it difficult to standardize optimal dosing concentrations in patients, as well as determine whether intake amounts correspond to clinical results. The lack of proper measurement and tracking of the compounds also prevents the ability to associate intake with any signs of toxicity. This challenge arises because MDPs are large, complex polysaccharides that are often poorly absorbed, making their detection in trace amounts within the bloodstream and tissues highly demanding for current analytical techniques.

Advances in the accurate and robust detection of MDPs in biological fluids and tissues have begun to address these challenges. Highly selective and sensitive immunoassays have allowed researchers to overcome some of these issues, although questions still remain regarding the detection of different polymer sizes [98]. Recent advances in tracking using isotopic labeling and the use of fluorescence have further assisted the identification and measurement of these molecules within the human body [98]. In using these advances in clinical testing, researchers can ensure that trials are designed to deliver compounds at optimal levels to patients, resulting in accurate and precise evaluation of marine polysaccharide efficacy. For example, attaching a fluorescent tag to a fucoidan molecule allows its movement and accumulation within specific tissues, like atherosclerotic plaques, to be visualized and quantified in animal models.

While, in certain circumstances, in vitro or animal studies of MDPs such as fucoidan did not result in toxicity even at high dosages for chronic time periods, the available clinical data on the safety of MDPs remain limited [99]. Most of the clinical data have involved short periods of intervention, with no standardized reporting of adverse events, or the long-term monitoring necessary to observe any potential rare or long-term toxicities [19]. This lack of adequate data may not be comprehensive enough to properly address risk–benefit analysis and obtain regulatory approval for therapeutic use in humans. It is also possible that potential long-term toxicities cannot be tested in the clinical timeframe of a trial and would necessitate long-term patient intervention in a clinical setting. This concern must be addressed if a patient were to be on a marine-derived polysaccharide supplement for a large period of time.

The variability in absorption, metabolism, and immune responses across species also represents a challenge. Marine polysaccharide compounds may result in toxicity in humans even though they did not exhibit this response in animal models [99]. There also need to be human clinical trials that focus on how the various structures and purity of marine polysaccharide molecules result in different therapeutic outcomes. To address this, proper techniques have recently been developed using fluorophores, isotopes, and sensitive analytical tools that can now track, visualize, and quantitatively measure accumulation and absorption in tissues and circulation in vivo [98]. Since a fucoidan might be metabolized differently by human gut bacteria compared to rodent gut bacteria, leading to distinct breakdown products that could have varied systemic effects or toxicities not observed in preclinical animal studies.

In addition to safety and efficacy concerns, clinical translation of MDPs is limited by the variability and discrepancies in product preparation, dosing regimens, and the endpoints examined in cardiovascular diseases [19]. As described earlier, different extraction techniques and methods of purification can result in a diversity of molecular compositions, which may alter therapeutic and toxicity results. There are also concerns surrounding the variable levels of contaminants, which have been found to impact the pharmacological and safety properties of these compounds. These factors could affect the interpretation of trial results across studies, hindering clinical progress and adoption. Different studies also examine different endpoints of cardiovascular disease; for example, one study may only assess plasma lipoproteins, while another assesses plasma cholesterol as well as systemic blood pressure.

In addition to inconsistent control groups and intervention durations, clinical trial methodologies in assessing the efficacy of marine polysaccharide compounds are limited by inconsistent product purity, dosing regimens, and endpoints, resulting in difficulties in comparing and synthesizing results across trials. While the need for well-designed and large-scale clinical studies is evident, one of the most important steps to further clinical progress of marine polysaccharide use to address atherosclerotic cardiovascular disease is the harmonization of clinical trial designs, specifically in terms of controls, endpoints, dosing, intervention duration, and subject populations [17]. Additionally, detection advances that enable more precise delivery and absorption measurement should be incorporated into study designs in order to confirm dosing and to link circulating compound levels with bioactivity [98]. This harmonization would mean that all trials evaluating a specific MDP for atherosclerosis would consistently measure, for example, LDL-C, HDL-C, triglycerides, and common inflammatory biomarkers like CRP, using standardized assays and time points, allowing for robust meta-analyses.

Overall, with advancements in detection and monitoring, MDPs offer a potent, novel, and alternative means to combat cardiovascular diseases. In order to best evaluate the translational potential, there need to be more well-constructed and large-scale, placebo-controlled clinical studies to truly evaluate the effectiveness and safety of MDPs to address atherosclerotic cardiovascular disease.

### 4.2. Bioavailability, Stability, and Pharmacokinetics

Since MDPs have a high molecular weight and are of an anionic nature, their oral bioavailability is generally poor and often inconsistent, owing to poor permeation through the intestinal epithelial cells passively or paracellularly [98]. For example, native fucoidan is poorly absorbed and has low bioavailability [99], thus limiting its biological effect. The absorption pathways for MDPs, viz., paracellular pathway, transcellular, and M cell–mediated transport, mostly depend on the size and charge of the molecules. Thus, large- and heavily charged molecules are less efficiently transported across the intestine, leading to low bioavailability [98]. This poor permeation occurs because the tight junctions between intestinal cells form a selective barrier, making it difficult for large, charged molecules to pass through the limited space of the paracellular pathway or to be actively transported across the cells.

Fucoidan-loaded nanoparticles have been shown to increase permeability across intestinal cell lines such as Caco-2 and also exert better bioactivity as compared to the free polysaccharide [99]. Molecular modifications that minimize the molecular weight and change the charge are helpful in the increased paracellular and transcellular transportation of polysaccharides [98]. Strategies that involve personalized approaches, considering individual recipient conditions like gut microbiota, general health, and genetics, might enable higher absorption and metabolism of MDPs [98]. For instance, encapsulating fucoidan within a chitosan nanoparticle can protect it from degradation and facilitate its uptake by endocytosis or enhanced paracellular transport, as observed in in vitro models.

Bioanalytical methods like ELISA, fluorescence labeling, and stable isotopes enable to monitor pharmacokinetic properties, tissue distribution of these compounds post-administration, absorption rate, bioavailability, distribution, metabolism, and elimination, dose–response studies, and the evaluation of the efficacy of polysaccharides in vivo [98]. Molecular weight, sulfation, and dosage determine the bioavailability and the subsequent biological effect of MDPs [98]. Higher-molecular-weight polysaccharides such as native fucoidan are not easily absorbed and have poor solubility in the physiological milieu, thus causing a limited biological effect, whereas lower-molecular-weight polysaccharides can be easily absorbed, thereby leading to enhanced bioactivity. The complex molecular composition and chemical configuration of MDPs create differences in pharmacokinetics and hence present a difficulty in developing therapeutic applications [98].

Since inflammation or changes in gut permeability can impact the absorption and efficacy of MDPs, tailoring the formulations as per the clinical profiles of the individual recipients would be critical for therapeutic efficacy [98]. Pharmacokinetic studies can improve the design and outcome of clinical trials by detecting the absorption, plasma concentration, and tissue distribution of MDPs after in vivo treatment, which is currently limited in several existing preclinical studies on MDPs [98]. These techniques are, however, capital-intensive and need to be used in routine preclinical studies for efficacy and standardization. Fucoidan-loaded chitosan nanoparticles have been shown to enhance absorption and uptake in intestinal cells [17]. This enhanced delivery occurs because nanoparticles can protect the MDP from enzymatic breakdown in the harsh gastrointestinal environment and facilitate its transport across biological membranes, ensuring that more of the active compound reaches its target.

MDPs formulated with nanoparticles may lead to improved intestinal permeability, prevent gastrointestinal degradation, maintain the structural and functional integrity, and prolong bioavailability and therapeutic potential of the polysaccharides in vivo and ex vivo. Encapsulation of fucoidan in chitosan nanoparticles enabled the slow and sustained release of the active drug and provided better protection and delivery of fucoidan in biological systems [99]. Nanoparticle-formulated fucoidan significantly ameliorated post-ischemic angiogenesis when compared to the native drug [99]. Several challenges exist for the development of the bioactive polysaccharides as a therapeutic agent, mainly in terms of batch-to-batch variability, the need for mass production, and limited clinical trials of these marine-derived agents [17]. This means that a highly sulfated, high molecular weight fucoidan, when orally administered, might be largely excreted unchanged due to its inability to cross the gut barrier, while a chemically modified or depolymerized version could show different absorption and elimination profiles.

Pharmacokinetics and systemic elimination of MDPs depend on their molecular characteristics and the route of administration. Orally administered fucoidan in rats has not demonstrated any toxicity and has been found to be safely excreted via the gastrointestinal tract, whereas mauran polysaccharides exhibited dose-dependent cytotoxic behavior in cell culture [99]. The ADME properties for most MDPs are unclear, as there are limited and inconsistent clinical and preclinical studies on pharmacokinetics. While native fucoidan might pass through the body without significant absorption, a nanoparticle-encapsulated fucoidan could accumulate in specific organs, necessitating long-term toxicity studies to rule out unforeseen adverse effects.

The pharmacokinetic and biodistribution data from the long-term in vivo studies in animal models are limited. Accumulation, off-target effect, and adverse events need to be ruled out by animal and human studies. This is critical as modified polysaccharides or nanoparticles can differ from the native polysaccharide in terms of ADME properties [99].

### 4.3. Variability in Structure–Function Relationships

MDPs differ considerably in their structures, which significantly influence their biological activities such as anti-inflammatory, antioxidant, and lipid-modulatory effects. This heterogeneity results from differences in monosaccharide composition, type of glycosidic linkages, molecular weight (MW), degrees of sulfation, and branching structures. This variability makes it difficult to correlate structure with biological activities, demanding a systematic approach to understanding structure–function relationships [9]. In some studies, higher sulfation levels in fucoidan were found to correlate with enhanced anticoagulant and anti-inflammatory activities, suggesting a significant influence of sulfation patterns in biological activity [100]. Lower-MW fractions, on the other hand, have been shown to have superior tissue penetration and better target specific bioactivity in regions of vascular inflammation [9]. Thus, understanding specific structure–activity relationships requires further investigation into how these structural characteristics interact with cellular and molecular targets. Studies comparing molecular characteristics with biological activities in ASCVD models are summarized in Table 2.

The monosaccharide composition of MDPs determines their therapeutic activity and therefore requires careful tuning. In fucoidan, the proportion of fucose, for instance, influences receptor interactions and downstream immunomodulatory and antioxidant mechanisms [9]. This underlines the need for standardized purification methods in preclinical development. For example, the β-1,3-glycosidic linkages present in laminarin are responsible for macrophage regulation and free radical scavenging [9]. As previously noted, many MDPs lack characterization regarding purity or bioactivity, especially those intended for oral use. The lack of standardized extraction methods has led to variability of bioactive profiles. Therefore, the oral delivery of bioactive polysaccharides still needs further characterization.

The lack of a standardized production procedure for these bioactive polysaccharides leads to their varying molecular compositions, which hinders direct comparison in most studies and poses an issue for future regulation and approval of these polysaccharides [9]. Also, variability across various polysaccharide preparations due to differences in extraction methods, source organism, and analytical methods results in variable safety and efficacy profiles. Structure variations in molecular charge and spatial configuration often dictate oral bioavailability. Largely sized polysaccharides with high negative charges have lower intestinal penetration, which demands new, more customized delivery systems [98]. These polymers could have a targeted bioavailability by going through pathways like transcellular absorption or receptor-mediated intestinal uptake.

Further structural features, such as branching degrees and acetylation, may play a critical role in stability and gastrointestinal absorption, influencing the final three-dimensional confirmation of bioactive MDPs [98]. They could influence their solubility and their stability in the digestive tract, changing the amount of them absorbed in an intact form into systemic circulation. Moreover, these modifications may increase polysaccharide resistance to degrading digestive enzymes in vivo and allow more intact structures to pass into systemic circulation. Thus, oral drug formulation and production for bioactive polysaccharides should be standardized. Some modifications to these polysaccharides, such as desulfation and low MW of chains, could lead to higher drug permeability, enhancing their therapeutic efficacy in vivo [98]. However, each modification may lead to novel toxicity and immune response that must be tested for in both in vitro and in vivo preclinical trials before going to clinical trials. For instance, a highly branched polysaccharide might offer more sites for enzymatic degradation, potentially reducing its stability and leading to lower systemic absorption of the intact molecule [102].

Comparative studies on pharmacokinetics have shown that slight differences in the marine polysaccharide structures result in significantly variable absorption rates and metabolic fates, which may affect clinical efficacy [98]. New methods in drug delivery are needed for bioactive MDPs. One area of growing interest is nanobased approaches. MDPs are a valuable source for nanoparticle development because of their intrinsic biodegradability, non-toxicity, stability, and easy availability, but the variability across batches and difficulties in achieving a specific, controlled drug release must be controlled [17]. Standardization in product and method development is a major area for development in this area of MDPs and the ability to better analyze and predict the structures of polysaccharides.

Bioactive MDPs have shown a new pathway for drug and treatment formulation. Several methods for improving drug release have been proposed, such as the synthesis of a polymer complex or conjugating polysaccharides on the nanoparticle surface. MDPs such as fucoidan are utilized in the manufacture of nanoparticle carriers, as the high bioavailability due to their negative charge and MW can be improved to deliver orally administered drugs into the systemic circulation [17]. However, the chance of these nanoparticle drug deliveries modifying immune responses and having additional toxicity can be high. It will be important to evaluate any new delivery to prevent unpredictable adverse immune responses and other unwanted biological outcomes [17]. Structure-related efficacy analysis and bioinformatics prediction are needed in marine-derived polysaccharide delivery methods, for which more detailed experiments with well-controlled quality should be performed. For example, creating a polymer complex by associating fucoidan with a positively charged protein could mask its negative charge, enhancing its interaction with the intestinal lining and improving absorption.

In vivo studies have also revealed large disparities between biological efficacy, safety, and dose administration across various experiments, indicating that standards for extraction procedures must be established. Thus, it is essential to identify methods that ensure product standardization by establishing quality control measures throughout the supply chain from the sea source to the delivery of these polysaccharides into the human body [9]. For example, different isolation and extraction methods can yield differences in MW, sulfation, degree of branching, and chemical purity that directly influence biological activities [101]. Variations in these factors may be responsible for the variations in activity observed across studies. Quality and yield may be influenced by different methods of production of MDPs. Thus, standards should be defined for their extraction process to ensure biological purity and consistency.

Significant anti-atherosclerotic properties were reported in a fucoidan study while another indicated negligible effect. These differences across various studies are due to differences in crude starting materials, analytical methodologies, and varying conditions in the different studies [9]. Better correlation and cross-validation of in vivo data must be addressed to help make clinical decisions. Also, there needs to be some level of openness, especially in the polysaccharide industry, to ensure quality of the product and the ability to aggregate results. Better product control by the polysaccharide production plants should be made to eliminate variances among batches. Standard production processes may be developed based on quality control via the chemical fingerprints of the final product, such as nuclear magnetic resonance (NMR), or mass spectrometry that measure the chemical makeup of compounds in the final polysaccharide product for quality assessment [98].

For the same algal species, environmental influences (light, salinity, and temperature) contribute to variations in polysaccharide yield and structure. Thus, better recording methods for the harvest source and season are critical [17]. Moreover, bioinformatics data and algorithms for polysaccharide structure–activity relationships are emerging, which may help identify and validate the link between marine-derived polysaccharide structure and biological functions, thus establishing clear molecular mechanisms and providing better predictability. The construction of new polysaccharides that will have increased therapeutic effects can be carried out rationally using this knowledge with the aid of structure–activity databases and high-throughput analytical platforms. The predictive models and bioinformatics analyses of structure–activity and toxicity may benefit the design and construction of novel polysaccharides for cardiovascular diseases [98]. Due to these complexities in defining polysaccharide structure–function relationships, a higher level of rigor in methods of analysis and the development of reliable production standards will be necessary if MDPs are to meet clinical relevance for cardiovascular disease therapies.

### 4.4. Safety Concerns

MDPs like fucoidan show a good safety profile in preclinical experiments. As an example, the sub-chronic oral administration of fucoidan up to 300 mg/kg body weight daily for 6 months in rodents did not reveal any significant toxicity, suggesting that some MDPs may be suitable for long use [99]. Nevertheless, no long-term clinical assessment in human populations has been performed so far, and it is necessary to conduct such a study in order to evaluate the long-term safety and possible toxicity of fucoidan in human bodies.

Although preclinical studies show a good safety profile for MDPs, inter-species differences in metabolism, immune responses, and many other cellular aspects pose a challenge when extrapolating animal data to humans. Rodents and humans do not metabolize polysaccharides in the same way; thus, they may have different biological effects in each organism, necessitating human clinical research in order to verify the safety of these compounds. Thus, it is necessary to confirm those initial assumptions, in order to guarantee and assure that these valuable findings in rodents are transferable to different human populations [99].

Even though information about appropriate dosing can be obtained from animal data, translation to humans may not be accurate; thus, the lack of longitudinal human clinical trials to assess the chronic consumption effects of MDPs in specific organs, like the liver and kidney, or in the immune system, is a limitation that needs to be addressed in order to ensure safe administration [19]. As long as long-term data on toxicity is deficient in human subjects, and because there is an absence of monitoring safety protocols and of any clear guidelines regarding the reporting of any adverse events, it is hard to assure the safety of using MDPs in the prevention and treatment of cardiovascular diseases [19]. Without long-term human studies, it is impossible to rule out subtle, cumulative toxicities that might manifest after years of exposure, such as mild liver enzyme elevations or a shift in immune cell populations, even if initial short-term trials show no issues.

Altered polysaccharide structure might create additional safety challenges. For example, mauran combined with reduced graphene oxide resulted in dose-dependent cytotoxicity in cell-based studies [99]. Because each derivative may have a particular toxic profile, depending on structural and chemical modifications, it may be necessary to consider a distinct evaluation of their safety. Changes to polysaccharide structure may drastically affect their safety profile, as modifications to molecular weight, sulfation degree, and addition of different functional groups can profoundly influence the interaction between them and the cells in human bodies, for example. Thus, structural characterization may be necessary to ensure that safety is not compromised while seeking for optimized bioactivity of these biopolymers [99]. This is because even a seemingly minor change, like increasing the negative charge through higher sulfation, can alter how a polysaccharide interacts with cell membranes or circulating proteins, potentially leading to unintended inflammatory or cytotoxic responses.

The encapsulation of MDPs in nanoparticles represents another way to improve their bioavailability and control over their release. On the other hand, potential complications may be created using this methodology due to the nanoparticle delivery method. The encapsulation of MDPs in nanoparticles can lead to unintended effects on membrane fusion, potentially compromising the safety of marine polysaccharide delivery to the targeted tissues or cells [98]. It is also important to take into consideration that MDPs may be contaminated with proteins or minerals (heavy metals, in the case of marine organisms) and may have residual solvent content (ethanol and isopropanol, for example) from the extraction processes [103,104]. These impurities are safety concerns because they may negatively interfere in biological functions of the body [19]. For instance, brown algae are known to accumulate up to 0.5 to 250 μg/g (dry weight) arsenic [105,106]. Some brown algae, like those in the *Sargassum* genus, can accumulate very high levels of inorganic arsenic (up to 83.7 mg/kg); others, like *Saccharina japonica* and *Undaria pinnatifida*, tend to accumulate lower amounts (0.02 to 0.24 mg/kg). Meanwhile, red and green algae can accumulate cadmium and lead [107,108,109]. Generally, green algae like *Chlorella* and *Ulva* have been shown to accumulate lead more effectively than cadmium. Specific concentrations vary widely, but some studies report lead accumulation in the range of 0.05 to 5.42 mg/kg dry weight, while cadmium levels range from 0.02 to 2.58 mg/kg dry weight. Since information related to marine polysaccharide toxicity in human beings is poor, safety assessment is highly necessary to validate preclinical findings [99].

Moreover, potential cytotoxicity in cells and potential influence of MDPs on vital organs should be evaluated, in order to predict or identify any undesired side effects in patients using these biopolymers [99]. Conflicting safety outcomes should be avoided by creating harmonization of experimental settings across future clinical studies [19]. However, even though MDPs are generally considered to be biodegradable and naturally non-toxic, it is important to be aware that there may exist the potential for unknown, long-term, or immunogenic side effects of these molecules [19]. For instance, while a specific MDP might not cause acute cell death, chronic exposure could induce subtle changes in gene expression or cellular signaling pathways in organs like the liver or kidney, potentially leading to long-term dysfunction.

Besides the lack of studies, the major technical bottleneck in the safety evaluation of MDPs arises from the challenge of quantifying them in the biological system. Polysaccharides are diverse structures and low in their concentration in systemic levels, making it difficult to accurately follow their biodistribution and metabolism, and thus leading to difficulties in addressing the chronic toxicity of the molecules [98]. Immunoassay, fluorescent labeling, and isotopic tracer techniques show promise as tools for overcoming these limitations and better quantifying MDPs in vivo. These methods also support safer administration of the required dosages [98].

Moreover, tissue accumulation and poor excretion of polysaccharides can result in adverse effects on the organs, particularly with long-term use. The need for accurate evaluation of distribution and excretion properties in the animal model can contribute to minimizing or eliminating the potential risk of their toxicity to the tissues [98]. Though many of these bioactive molecules have low toxicity, the variations in their composition, extraction conditions, contaminant sources, and biological activities may compromise their use as a health-promoting supplement and therapeutic agent. For example, impurities that come along with the biopolymer from the marine organisms may affect their function and the safety [19]. If certain MDPs, even those generally considered safe, accumulate in a particular organ over time, it could disrupt cellular processes or trigger localized inflammation, leading to chronic organ damage that would not be apparent in short-term studies.

The commercialization of several products containing polysaccharides as supplements or drugs may indicate their safety, although each class of polysaccharide, derived from specific sea organisms, or from particular fractions and with different treatments, represents a different chemical and pharmacological profile [19]. Each modified marine polysaccharide can produce a unique profile in the body. Hence, no generalization can be made for the clinical safety of these molecules. Monitoring immunogenicity and batch-to-batch contaminants and uniformity are necessary steps in reducing their toxicity risk to patients [99]. Future clinical investigations are needed; moreover, in order to further advance the safety assessment of MDPs, the need to develop and test accurate detection methods is fundamental [98].

## 5. Future Perspectives and Strategic Opportunities

Optimizing the oral bioavailability of MDPs is a key step in maximizing their cardiovascular protective effect. The main problem in their clinical application is poor intestinal absorption. Both their high molecular weight and high negative charge hinder their systemic exposure and bioactivity. Their large size and numerous anionic sulfate groups of fucoidans restrict their passage across the tightly packed epithelial cells of the intestinal barrier, limiting passive diffusion and active transport mechanisms, thereby resulting in minimal systemic uptake and, consequently, low bioavailability [110]. This poor absorption is further compounded by enzymatic degradation in the gastrointestinal tract, contributing to their reduced systemic exposure and diminished therapeutic efficacy for applications requiring internal systemic action. Although delivery systems such as nanoparticles have shown better permeability and bioactivity, such as fucoidan-loaded nanoparticles having better intestinal absorption across the Caco-2 cell monolayer and improved exposure in vivo, these approaches may have safety, biocompatibility, and regulatory issues [17,98]. Long-term safety and toxicity studies, as well as new regulations, are needed to ensure immune activation or systemic retention of nanoparticles, which may limit clinical acceptance of this novel delivery system. Furthermore, controlled depolymerization and customized sulfation modification strategies also optimize the size and charge, and then promote interaction with paracellular, transcytosis, and M cell pathways, thereby improving bioavailability [98]. This kind of strategy can also prevent polysaccharides from degradation and clearance in the gastrointestinal tract, prolonging circulation time and allowing for sufficient accumulation in the vascular tissues, thus improving the cardiovascular protective effect.

Despite the promising ability of nanoparticles to enhance the oral bioavailability and vascular targeting of marine-derived polysaccharides, their clinical translation is hindered by unresolved safety and biocompatibility concerns. Nanoparticles, especially those with prolonged circulation or high surface reactivity, may trigger unintended immune responses, including complement activation and cytokine release [111]. There is also growing concern over long-term accumulation in tissues such as the liver, spleen, and lymph nodes, which could lead to chronic toxicity or organ dysfunction [112]. Polymer-based systems like chitosan-fucoidan nanoparticles may offer improved mucosal adhesion and lymphatic uptake, but risk immunogenicity due to their cationic surface [113], while lipid-based nanoparticles often demonstrate better biocompatibility and biodegradability but suffer from lower encapsulation efficiency and stability [114]. A critical balance between enhancing delivery and minimizing biological reactivity is essential for advancing these formulations toward clinical use, especially in chronic conditions such as ASCVD, which require long-term treatment.

In vivo studies evaluating the nanoparticle-mediated delivery of MDPs in ASCVD models remain limited, but the existing evidence suggests that formulation type plays a significant role in determining therapeutic outcomes. Polymer-based nanoparticles have shown promise in reducing plaque burden, likely due to sustained release and enhanced endothelial uptake, but they may induce local tissue irritation or off-target immune responses [115]. In contrast, lipid-based carriers such as liposomes or solid lipid nanoparticles can improve bioavailability and reduce oxidative stress in atherosclerotic lesions, while maintaining a favorable safety profile [116]. Some formulations have demonstrated improved targeting of inflamed vascular regions, enhanced macrophage modulation, and even partial plaque regression, highlighting the potential of tailored nanocarrier systems in ASCVD therapy [117,118]. However, each delivery system carries distinct efficacy-safety trade-offs that must be optimized based on the polysaccharide’s properties, the route of administration, and the intended therapeutic target. A deeper understanding of the interactions between nanoparticle physicochemical characteristics and the pathophysiology of atherosclerosis will be critical for designing safe, effective, and scalable delivery platforms.

Developing precise pharmacokinetic and biodistribution profiling methods is crucial for translatability to humans. Current detection and profiling techniques of MDPs are still in their early stages. Challenges such as difficulty in detecting or tracking them due to their complex chemical structure, unknown pathways of absorption, biodistribution, metabolism, and elimination make the evaluation in preclinical studies inadequate [119]. Development of improved analytical techniques, such as fluorescence, radioactivity, or immunological labeling, and isotope tracer technologies, would increase the precision for quantifying absorption rates, tissue distribution, metabolism, and elimination profiles [98]. Accurate monitoring of these pharmacokinetic parameters would allow better dose optimization, efficacy evaluation, and toxicity prediction, which are essential in clinical applications. Additionally, advances in analytical methods enable researchers to better understand the mechanism of action by identifying and profiling their bioactive metabolites. Pharmacokinetic and biodistribution studies will help to provide critical data for clinical trial design and endpoint analysis for approval and marketing of these MDPs in future clinical practice.

Standardizing extraction, characterization, and safety profiles of MDPs will reduce inconsistent outcomes. Currently, the crude methods of extracting and processing MDPs from organisms of different sources cause a significant variation in their structure and bioactivity from batch to batch, and therefore affect their application in drug research and product development. The different extraction methods and source materials lead to variations in critical parameters, such as molecular weight, sulfate content, and purity, which all affect the anti-atherosclerotic activities [17,42]. Furthermore, different standards of extraction, characterization, and quality control greatly hamper both inter- and intralaboratory reproducibility. Using validated characterization techniques such as nuclear magnetic resonance spectroscopy and chromatographic profiles to ensure quality parameters will support manufacturing consistency of product composition. Toxicity assessment, as well as the harmonized safety protocol compliant with international requirements, is another factor that limits their use for cardiovascular protection [19]. Immunogenicity and potential heavy metal contamination, as well as the lack of standardization in assessing their toxicity, also negatively impact pharmaceutical application [120]. Therefore, well-validated standardization and characterization strategies are required to provide quality assurance of these promising marine-derived products.

Determining structure–activity relationships will pave the way for therapeutic designs of polysaccharides. The biological activity of polysaccharides, such as anti-inflammatory and antioxidant functions, are greatly dependent on molecular structure [42,98]. High-throughput screening and computational simulation can improve structural optimization with limited experiments in an unbiased manner and allow rational designing of high-quality lead compounds, which in turn would lead to new drugs for combating atherosclerotic cardiovascular disease. In fact, such correlations between chemical characteristics and in vitro bioactivities have been observed in previous reports, such as specific sulfation patterns and branching patterns being associated with improved lowering of lipid levels, and inhibiting NF-κB signaling. These results suggest that the structure–activity relationships may differ in terms of specificity, thus leading to the possibility of developing polysaccharides with individualized effectiveness [121]. The use of bioinformatics and prediction model development to achieve personalized medicine approaches for polysaccharides should be future research areas. Analysis of these structure–activity relationships also provides us with intellectual property and technological protection for the novel polysaccharides. However, this method requires expertise in organic chemistry, protein chemistry, and bioinformatics, as well as high-throughput screening facilities.

Interdisciplinary collaboration is vital in order to overcome the challenges associated with translating MDPs into anti-atherosclerotic therapies. MDPs are inherently a highly interdisciplinary area involving expertise from marine biologists, pharmacologists, chemists, and clinicians. Marine biologists contribute their expertise to identify the suitable species, the geographical location, and seasonal patterns that would enable maximum and sustainable harvest of these compounds for large-scale extraction. Pharmacologists and chemists are essential for effective extraction of these compounds, as well as for the characterization and elucidation of structures. Chemists and pharmacologists also bring knowledge of their activity and mechanism, as well as optimization for delivery, to ensure the translation and development of polysaccharide therapeutics [17,42]. Last but not least, clinicians play a critical role in leading the efforts to move translational research to improve benefits to patients with cardiovascular disease. Interdisciplinary collaboration is the key to successful advancement of MDPs as therapeutics for cardiovascular protection. Clinical trials are critical to provide safety and efficacy data and should utilize patient-oriented clinical endpoints. Furthermore, interdisciplinary collaborative teams would improve novel delivery design and promote preclinical validation to advance new treatments from ocean organisms to the patient bedside and support the overall discovery process.

To achieve robust interdisciplinary collaboration, actionable steps include establishing joint industry–academia consortia for GMP-compliant marine-derived polysaccharide (MDP) production, fostering consistent quality and scalability. Additionally, developing shared, open-access databases for structural characterization and bioactivity data is crucial, enabling comprehensive structure–function analyses. Finally, implementing standardized preclinical and clinical trial protocols across research institutions will ensure data comparability and accelerate regulatory approval.

## 6. Conclusions

The therapeutic promise of marine-derived polysaccharides for atherosclerotic cardiovascular disease is clear from extensive preclinical evidence demonstrating their anti-inflammatory, antioxidant, lipid-modulatory, antithrombotic, and endothelial-protective activities. However, their clinical translation is hindered by structural heterogeneity, poor and variable oral bioavailability, limited pharmacokinetic and toxicity data, and inconsistent extraction and characterization methods. Addressing these barriers through interdisciplinary collaboration—standardizing molecular structures, implementing advanced delivery systems (e.g., nanoparticles), and conducting well-designed human trials with defined endpoints—will be essential to validate safety, optimize dosing, and establish efficacy. With these efforts, MDPs could emerge as safe, multi-targeted adjuncts or alternatives to current therapies, unlocking under-utilized marine resources for nutraceutical and pharmaceutical development.

## Figures and Tables

**Table 2:** Structure–activity relationships of marine-derived polysaccharides in ASCVD models.

Polysaccharide Type	Structural Feature	Observed Biological Activity	Citation
Fucoidan (brown algae)	High sulfation (≥25%)	↑ Anticoagulant and anti-inflammatory activity via NF-κB inhibition and enhanced antithrombin III binding	[100,101]
Fucoidan	Low MW (3–10 kDa)	↑ Endothelial repair, ↑ endothelial nitric oxide synthase (eNOS) expression	[80,101]
Ulvan (green algae)	Rhamnose-rich, moderate sulfation	↓ TNF-α and IL-6, ↓ VCAM-1 expression in vascular tissues	[42,100]
Laminarin (brown algae)	β-1,3-glycosidic linkages	↑ Macrophage regulation, ↑ ROS scavenging	[9]
Chitosan oligosaccharide	Low MW (≤5 kDa), high degree of deacetylation	↑ Bile acid excretion, ↓ serum LDL-C and total cholesterol	[9]
Carrageenan (red algae)	High MW and high sulfation	↑ Platelet aggregation inhibition but also ↑ bleeding risk	[42]
Fucoidan	Low sulfation (<10%)	↓ Anticoagulant activity; reduced inhibition of thrombin and factor Xa	[101]

↑ increase and ↓ decrease.

## Data Availability

No data was produced from this study; all data used is contained in this article and published paper in the references.
